# Runnels mitigate marsh drowning in microtidal salt marshes

**DOI:** 10.3389/fenvs.2022.987246

**Published:** 2022-11-03

**Authors:** Elizabeth B. Watson, Wenley Ferguson, Lena K. Champlin, Jennifer D. White, Nick Ernst, Habibata A. Sylla, Brittany P. Wilburn, Cathleen Wigand

**Affiliations:** 1 Department of Biodiversity, Earth and Environmental Science, Academy of Natural Sciences of Drexel University, Philadelphia, PA, United States; 2 Save the Bay, Providence, RI, United States; 3 Rhode Island National Wildlife Refuge Complex, Charlestown, RI, United States; 4 Atlantic Coastal Environmental Sciences Division, US Environmental Protection Agency, Narragansett, RI, United States

**Keywords:** runnel, sea level rise, restoration, salt marsh, climate change, mitigation and adaptation, remote sensing, ditching

## Abstract

As a symptom of accelerated sea level rise and historic impacts to tidal hydrology from agricultural and mosquito control activities, coastal marshes in the Northeastern U.S. are experiencing conversion to open water through edge loss, widening and headward erosion of tidal channels, and the formation and expansion of interior ponds. These interior ponds often form in high elevation marsh, confounding the notion applied in predictive modeling that salt marshes convert to open water when elevation falls below a critical surface inundation threshold. The installation of tidal channel extension features, or runnels, is a technique that has been implemented to reduce water levels and permit vegetation reestablishment in drowning coastal marshes, although there are limited data available to recommend its advisability. We report on 5 years of vegetation and hydrologic monitoring of two locations where a total of 600-m of shallow (0.15–0.30-m in diameter and depth) runnels were installed in 2015 and 2016 to enhance drainage, in the Pettaquamscutt River Estuary, in southern Rhode Island, United States. Results from this Before-After Control-Impact (BACI) designed study found that runnel installation successfully promoted plant recolonization, although runnels did not consistently promote increases in high marsh species presence or diversity. Runnels reduced the groundwater table (by 0.07–0.12 m), and at one location, the groundwater table experienced a 2-fold increase in the fraction of the in-channel tidal range that was observed in the marsh water table. We suggest that restoration of tidal hydrology through runnel installation holds promise as a tool to encourage revegetation and extend the lifespan of drowning coastal marshes where interior ponds are expanding. In addition, our study highlights the importance of considering the rising groundwater table as an important factor in marsh drowning due to expanding interior ponds found on the marsh platform.

## 1 Introduction

**FIGURE 1:**
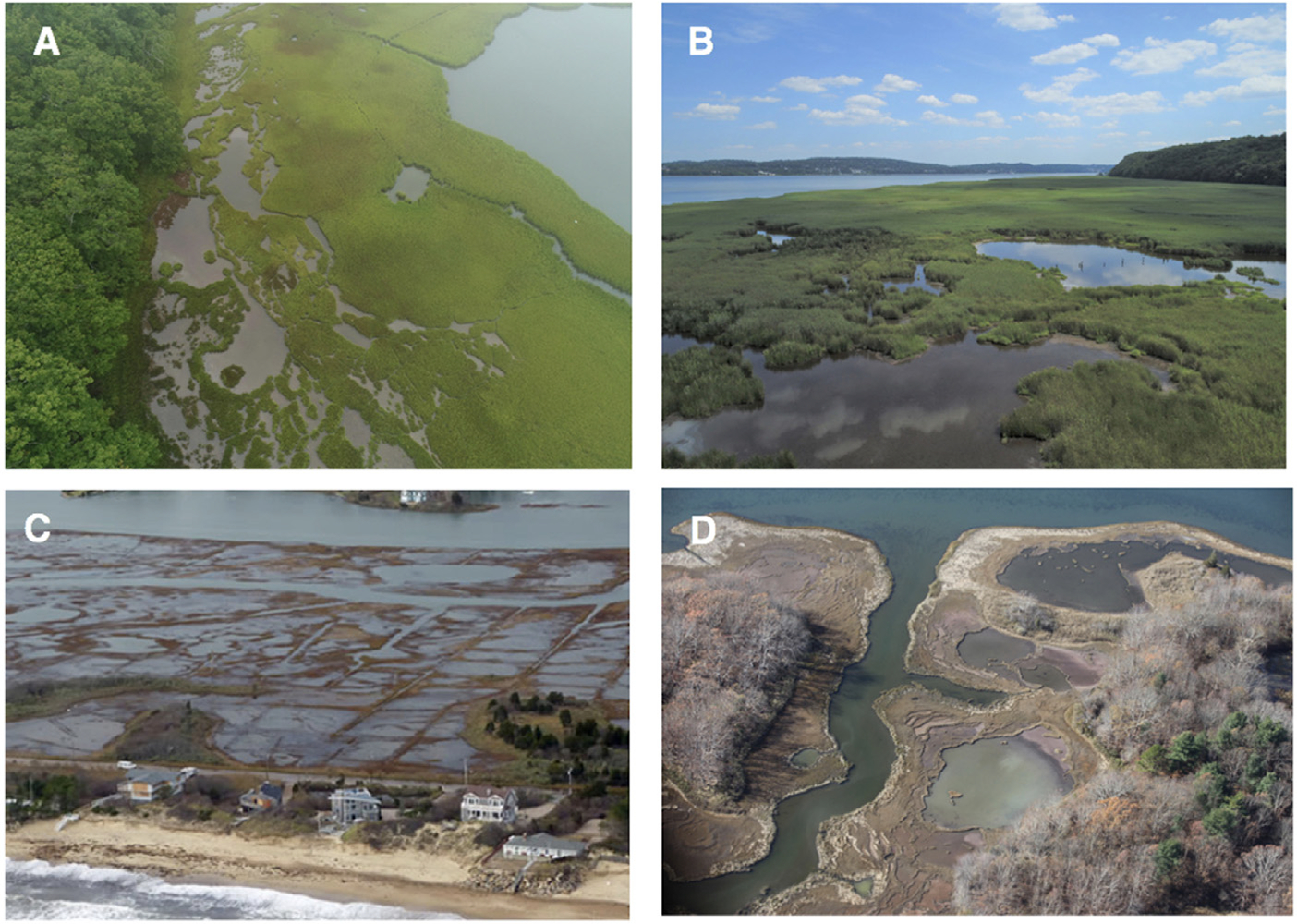
Examples of locations where ponding is contributing to coastal marsh habitat loss in New York and New England: **(A)** Bass Creek, Shelter Island, NY where ponds are expanding at the marsh-upland border; **(B)** Piermont Marsh, NY where ponds on the marsh interior are expanding; **(C)** Winnapaug Pond, RI where ponds have formed in grid-ditched marsh islands; and **(D)** the Pettaquamscutt River Estuary, RI, the focus of this study, where the marsh platform is dominated by large shallow ponds. Photographs courtesy of (A/B) Johannes Krause/Florida International University, **(C)** Jonathan Stone/Save The Bay, and **(D)** Greg Thompson/USFWS.

Photogrammetric analysis has shown that coastal salt marsh loss in New York and southern New England (United States) over the past 40 years has occurred at rates of 5% per decade (Smith, 2009; Berry et al., 2015; Watson et al., 2017; Krause et al., 2020). These coastal salt marsh losses have occurred primarily due to symptoms associated with accelerated relative sea level rise (SLR), such as marsh edge loss due to erosion, widening and expansion of the tidal channel network, and the formation and coalescence of interior ponds (Figure 1; Hartig et al., 2002; Mariotti, 2020). Crab herbivory, fungal pathogens, and nutrient pollution have also been implicated as stressors (Deegan et al., 2012; Elmer et al., 2013; Smith and Green, 2015; Raposa et al., 2018). Another important exacerbating factor to the formation of open water areas has been modifications to tidal hydrology, including agricultural embankments and ditching (Burdick et al., 2020; Smith et al., 2021). However, the recognition that marsh loss—as a symptom of climate change—is already occurring has led to a shift in how coastal land managers are approaching restoration and conservation (Watson et al., 2017; Wigand et al., 2017).

Over past decades, the restoration of coastal salt marshes in New York, New Jersey, and New England has focused on reestablishing tidal hydrology to restore ecosystem functions lost when marshes were filled and diked, and to reverse the invasion of *Phragmites australis. Phragmites australis* is a cryptic invasive species (Saltonstall, 2002). Its increased abundance over past decades has been associated with negative effects to vegetation and bird diversity (Chambers et al., 1999), and it is one of the most aggressively managed plants in the United States (Rogalski and Skelly, 2012). In addition to *Phragmites-*removal, restoration projects traditionally have focused on the removal of tidal restrictions and dikes to restore or amplify tidal exchange, the removal of fill to reduce elevations, and hydrological alterations to restore water to the landscape, such as plugging the extensive ditches constructed during the Works Progress Administration, or the direct excavation of ponds (Roman et al., 2002; Vincent et al., 2013; Powell et al., 2020). However, support for such approaches is waning because such actions have the potential to compromise the long-term stability of coastal habitats and survival of wildlife given accelerations in SLR. For example, a recent study that focused on the effects of restoration to the saltmarsh sparrow (*Ammodramus caudacutus*), which is a marsh-breeding bird considered globally vulnerable to extinction, found that *Phragmites* removal and tide restoration negatively impacted sparrow reproductive success, as it created habitats unsuitable for sparrow nesting (Elphick et al., 2015). As such, restoration and conservation of coastal marshes is shifting away from increasing inundation towards extending the lifespan of drowning marshes.

A project that exemplifies this shift in priorities has been the reconstruction of drowning and eroding marsh islands in Jamaica Bay, NYC (Campbell et al., 2017). The Jamaica Bay restoration project used 190,000 m^3^ of dredged sediment in combination with planting over 600,000 plant plugs to build the elevation of several disappearing marsh islands, thereby lengthening their lifespan (Messaros et al., 2010). In addition to this work in NYC, which began in 2003, a series of projects were constructed following Superstorm Sandy that focused explicitly on the dual goals of community and coastal marsh ecosystem resilience. Such approaches included the beneficial use of sediment placement to build marsh elevation, shoreline protection through installation of living shorelines, and facilitation of upland migration of marsh habitats (Wigand et al., 2017; VanZomeren et al., 2018; Weis et al., 2021).

One of the newer and less well known techniques that has been piloted over the past decade to extend the lifespan of drowning marshes is the strategic use of runnels, or channel extension features, to drain areas of ponded water found on the marsh platform with the goal of encouraging coastal marsh revegetation (Besterman et al., 2022; Perry et al., 2022). Ponds can be natural marsh features that provide important habitat functions (Adamowicz and Roman, 2005; Smith and Niles, 2016); however, the formation and expansion of shallow depressions filled with standing water that do not drain during daily tidal flow on the marsh platform may also contribute to permanent marsh loss (Mariotti, 2016). This is particularly true when this impounded water is associated with hydrologic modifications, such as agricultural embankments or extensive grid-ditching networks which are often associated with spoils, or where shallow ponds are experiencing runaway expansion caused by wind-wave erosion (Mariotti, 2020) (Figure 1). Also, some impounded water areas are a legacy of altered hydrology and agricultural embankments (Adamowicz et al., 2020).

Ponds form on the marsh landscape where the water table is at or above the marsh surface. Ponds are often described as transitory features, as their formation and capture by the tidal channel network and subsequent drainage has been recognized (Collins et al., 1987; Wilson et al., 2014; Supplementary Figure S1). Cyclic processes of pool formation, enlargement through expansion and mergence, drainage *via* tidal creek incision and recolonization by marsh vegetation has been described for New England, specifically in Maine and Massachusetts (Wilson et al., 2009, 2014), as well as southern New England and the Mid-Atlantic (Smith and Pellew, 2021). Observations of pond drainage resulting from creek incision demonstrate that connecting a pond with the tidal marsh drainage network occurs naturally and results in plant recolonization (Supplementary Figure S1; Smith and Pellew, 2021). The construction of runnels (typically 0.15–0.3 m wide and in depth) is designed to mimic this natural process of pond channel capture that occurs in tidal marshes. However, in microtidal estuaries, this process may take several decades or not occur, as these systems lack robust tidal exchange. Reversing marsh drowning through the installation of runnels can mimic and accelerate the natural process of drainage that occurs following pond capture by the tidal channel network, and can thus be an important tool to counteract marsh drowning (Taylor et al., 2020; Weis et al., 2021; Besterman et al., 2022).

**FIGURE 2:**
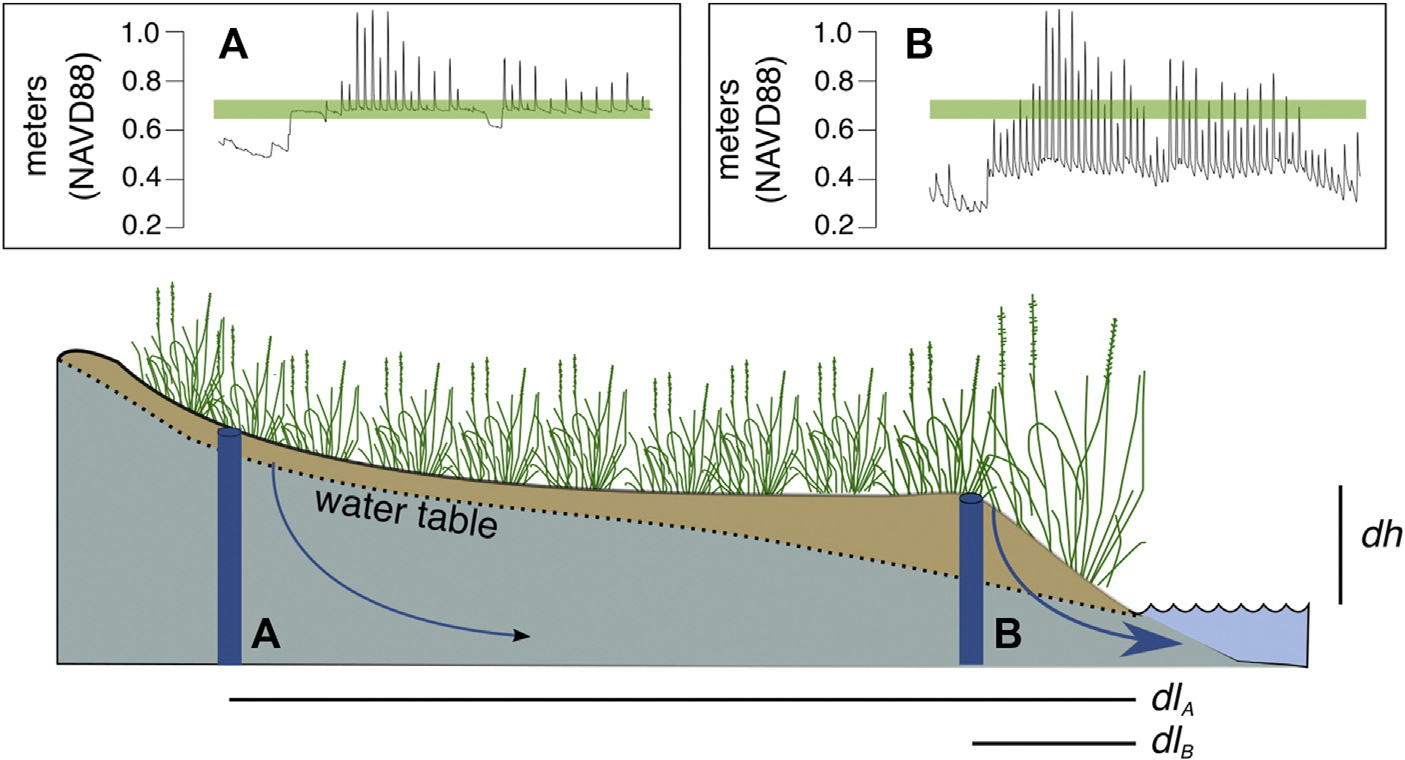
In coastal marshes, the water table tends to be close to the marsh surface (indicated by the green line) in the marsh interior, i.e., 30 m+ from the marsh edge, demarcated in this figure as location **(A)**. Along the edge of the marsh where it intercepts the tidal creek, the water table drops to much lower elevations at low tide, demarcated in this figure as location **(B)**. This is because through-marsh drainage is proportional to the product of soil hydraulic conductivity (*K*) and the hydraulic gradient (*dh*/*dl*). Values for the hydrologic gradient are highest adjacent to the tidal channel but decrease proportionally with distance (*l*) from the creek edge. This explains why drainage is reduced at location **(A)** in comparison with location **(B)**. Water table elevations were measured in spring of 2016 at Colt State Park, Bristol, Rhode Island (41.6769°N, 71.2985°W).

Connecting a marsh pool with a tidal creek can encourage revegetation through promotion of surface drainage (Wilson et al., 2014), but effects on sub-surface drainage are previously unstudied. Generally, ponds occur on the marsh platform due to the high and invariable water table, while the water table adjacent to tidal creeks is more variable (Montalto et al., 2006). Areas adjacent to tidal channels experience high variability in the level of the water table—often decimeters above the marsh surface at high tide and decimeters below the marsh surface at low tide. Adjacent to tidal channels, the low tide water table is typically far below the marsh surface due to the enhanced hydraulic gradient found at the channel edge (Figure 2). Conversely, marsh ponds usually occur on the marsh platform, where the hydraulic gradient is much smaller; and the water table tends to sit close to the marsh surface and vary little diurnally (Montalto et al., 2006). This spatial variability in water table dynamics and through-marsh groundwater flow contributes to the ecological zonation apparent in salt marshes, with larger growth forms of plants found on channel edges where the marsh supports better drained soils, and stunted growth forms in the marsh interior, where the water table is often stagnant and soils are exposed to salinity and sulfide accumulations due to poor drainage (Nuttle, 1988; Wilson et al., 2015). By installing runnels that drain water off the marsh surface, enhanced sub-surface drainage may extend more broadly across the marsh platform.

The purpose of the present study was to ascertain whether the construction of channel extension features (in 2015 and 2016) has contributed to vegetation reestablishment and enhanced drainage at a Rhode Island estuary where pond formation and expansion has contributed to marsh vegetation loss over the past century (Watson et al., 2014; Watson et al., 2017). A Before-After Control-Impact (BACI) study design (Stewart-Oaten et al., 1986) was used to compare vegetation coverage and water table dynamics. Analysis of high-resolution satellite imagery and vegetation surveys were used to establish vegetation trends, and well installation and groundwater monitoring were implemented to establish whether runneling lowered groundwater levels. Results of this study improve our understanding of marsh groundwater dynamics, as well as the advisability of marsh drainage enhancements as a tool to build coastal ecosystem resilience to SLR.

## 2 Materials and methods

### 2.1 Study site

**FIGURE 3:**
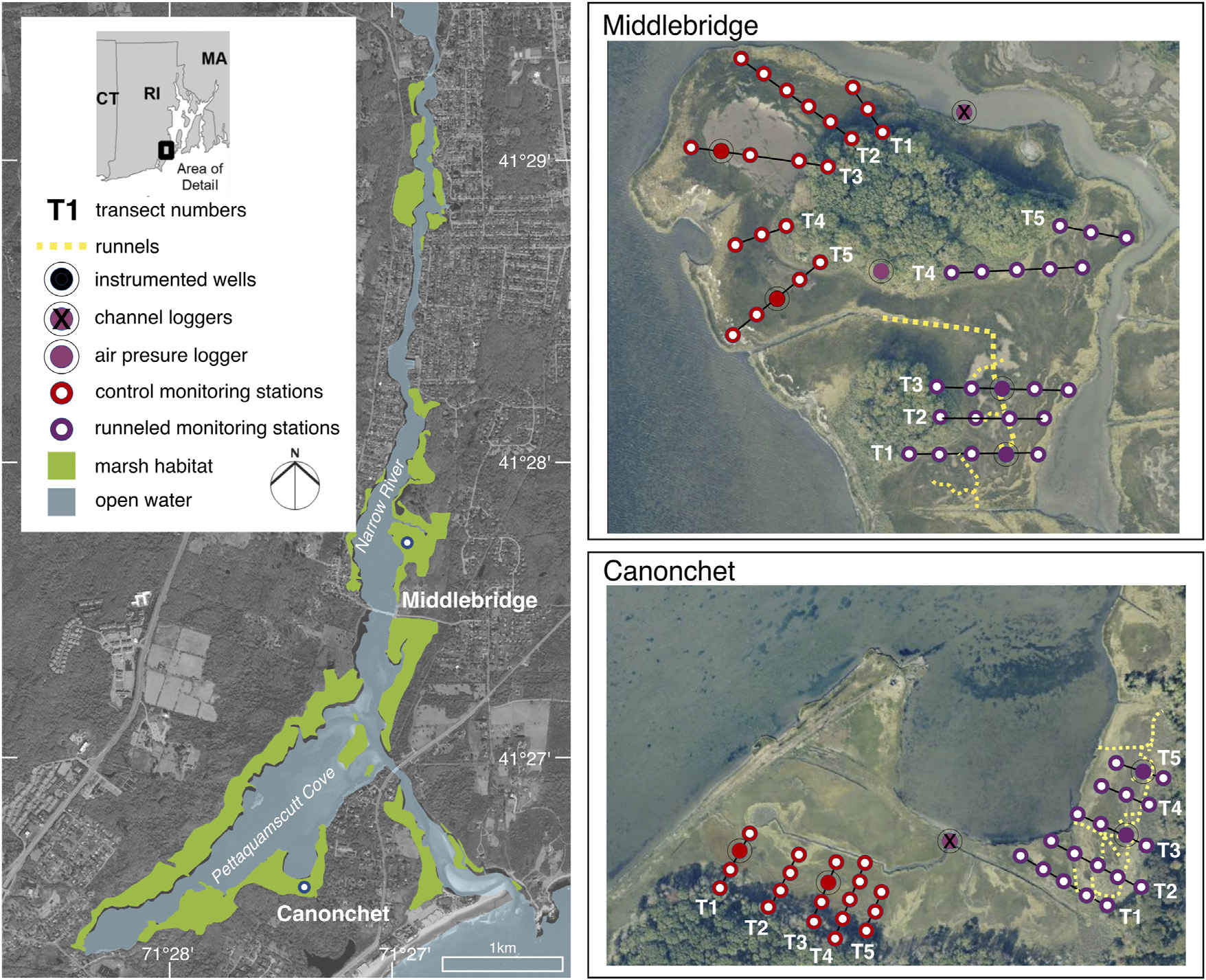
Location map showing the Narrow River Estuary, including runneled and control areas at Middlebridge and Canonchet. Vegetation monitoring transects are depicted as T1–T5. Red areas are control areas, purple are runneled areas. Groundwater wells monitored (focal wells) are depicted by a circle with a solid fill.

Research was conducted at the Pettaquamscutt River Estuary (PRE) (also called the Narrow River Estuary), part of the USFWS John H. Chaffee National Wildlife Refuge, Narragansett, Rhode Island (41.4547°N, 71.4533°W). The PRE is a 15 km-long river/estuarine system comprised of a tidal inlet, coastal estuary, and two kettle ponds, spanning the towns of Narragansett, North Kingstown, and South Kingstown, RI, United States (Figure 3). The PRE drains a 35 km^2^ watershed, of which 35% is classified as developed, and it supports a variety of diverse estuarine habitats, including eelgrass beds, estuarine channels, tidal mudflats, and salt marsh. Water column salinity ranges from 24 to 27‰ (Greening et al., 2018). Based on this study, we found the average diurnal range of tide to be 0.43 m at our research sites. The two focus areas, Canonchet and Middlebridge (Figure 3), varied somewhat in their inundation patterns. Canonchet has a slightly higher elevation and was found to be inundated 7.1% of the time, while Middlebridge was found to be inundated 14% of the time.

Runnels were constructed as part of resilience restoration actions occurring during 2015–2018, which included installation of living shorelines, dredging and sediment deposition to raise marsh elevation, and runneling to restore marsh hydrology in parts of the PRE (Wigand et al., 2017; Perry et al., 2022). This study focuses only on effects of runnels at Canonchet and Middlebridge; sediment addition and living shoreline installation were undertaken outside our area of study. Installation of these channel extensions was chosen as a restoration action because a substantial amount of habitat at the PRE was considered degraded due to a lack of drainage, and marsh elevation change data suggested that marsh accretion was not keeping pace with rates of SLR (Watson et al., 2014; Raposa et al., 2017). Improving drainage was a specific concern due to the focus on restoring high marsh vegetation to support marsh breeding birds (Berry et al., 2015), and because an analysis of vegetation distribution patterns suggested high marsh vegetation was controlled by drainage rather than by elevation (Watson et al., 2014). Runnels were constructed in two separate areas: an area south of the tidal inlet which we refer to as “Canonchet” due to its proximity to Canonchet Farm, and a northerly site we refer to as ‘Middlebridge’ due to its location north of Middlebridge Road (Figure 3). A total of 605 m of runnels (476 m at the Canonchet site; 129 m at the Middlebridge site) were constructed in spring of 2015 and 2016 by hand and using a low ground pressure excavator, connecting ponded areas with existing ditches or tidal channels (Figure 3). The runnels were 0.15–0.30 m in diameter and in depth. The peat excavated was retained on the marsh platform, but outside of the footprint of the vegetation transects. Although needed permits vary by jurisdiction, to complete this work, permits were obtained from the Coastal Resources Management Council (a State Agency), and the US Army Corps of Engineers.

### 2.2 Restoration monitoring

A Before-After Control-Impact (BACI) study design was used to study the effects of runnel excavation (Stewart-Oaten et al., 1986), with pre-construction plant surveys conducted in 2014. A series of transects were established in summer of 2014, with vegetation monitoring stations established every 12–20 m and groundwater monitoring stations installed every 25 m across the transects (Figure 3).

#### 2.2.1 Vegetation

Vegetation monitoring was conducted in 2014–2019 and coincided with peak biomass (mid-August through mid-September) (Table 1). Plant species composition and abundance along transects were measured along five transects per site randomly situated in each experimental unit traversing the marsh from creek or open water to the upland edge (Roman et al., 2001). Vegetation was sampled in 1-m^2^ plots located along transects, yielding a total of 20 plots each along the five transects. Using the point-intercept method (Roman et al., 2001) vegetation at 50 points in the plot was recorded. These data were used to calculate percent cover for each species; values may sum to excess of 100% where dowels touched multiple plants.

Changes in vegetation cover from 2014 to 2019 were also monitored using image classification and spectral indices calculated from georeferenced satellite imagery (Table 1). Imagery of the study sites was collected from spring and summer months. Habitat classification of annual satellite imagery was performed using a maximum likelihood classification algorithm using ENVI version 5.4 (Exelis Visual Information Solutions, Boulder, Colorado, United States) and ArcMap version 10.2.2 (Environmental Systems Research Institute, Redlands, CA, United States) (Otukei and Blaschke, 2010). Classification categories included fully vegetated, patchy vegetation, and open water. Annual satellite imagery was further examined using the normalized difference water index (NDWI) and a modified bare soil index (BSI) (Gao, 1996; Azizi et al., 2014). These band indices were calculated as:

(1) $$NDWI=\frac{\;green\;-\;NIR}{\;green\;+\;NIR}$$

(2) $$BSI=\frac{[red\;+\;blue]\;-\;green\;}{[red\;+\;blue]\;+\;green\;}$$

where *NDWI* refers to the normalized reflectance difference between the green and near-infra red [*NIR*] spectral bands (510–581 nm and 780–920 nm, respectively), and the *BSI* refers to the ratio of the difference between the sum of the red (655–690 nm) and blue (450–510 nm) spectral reflectance and the green (510–581 nm) reflectance to the sum of the red, blue, and green reflectances. *NDWI* was used to estimate the amount of open water habitat, while *BSI* was used to determine the area of bare soil.

#### 2.2.2 Water table and porewater salinity

The water table was monitored during the years 2014 through 2018 using shallow (0.70-m depth; 10 cm diameter; screened across the full 0.7-m length) wells installed along transects (Figure 3). At Canonchet and Middlebridge, two wells each were instrumented and monitored in control and runneled areas, as well as in the tidal channel (10 total; 4 runneled, 4 control, 2 channel). Wells were instrumented with pressure transducers during fall of each year (Table 2), with water levels measured at a 15-min interval, with reference water levels measured upon deployment and removal. Hydraulic conductivity of the marsh sediments was measured in spring and fall of 2020 from bail down tests performed in the four focal wells at each site (Figure 3) where the water was removed from the well using a pump and the rate at which the water rose was recorded (Hvorslev, 1951).

Porewater salinity monitoring was conducted biweekly at low tide during the growing season following the protocol developed by Roman et al. (2002). Porewater was taken from 15 cm below the marsh surface using a stainless-steel probe, near the PVC wells mentioned above. The salinity of the porewater was measured with a refractometer. If water was not able to be collected at 15 cm depth, the probe was inserted to 30 cm, then 45 cm if necessary. If porewater was not able to be collected in this manner, water was taken directly from the well.

#### 2.2.3 Data analysis

##### 2.2.3.1 Vegetation comparisons between treatments

Comparison of vegetation cover (bare and dominant plant species), species richness, and the Shannon Diversity Index (SDI) were carried out using a repeated measures, multi-factor ANOVA, with transect nested within the treatment variable. The effects of time (before, 2014; during, 2015–2016; after, 2017–2019) and treatment (runneled vs. control) were examined for each site (Canonchet and Middlebridge). Evaluations of treatment significance were performed using transect variability as the associated error term, and evaluations of the time by treatment interaction significance and individual pairwise comparisons within the context of that interaction were done using transect by time variability as the associated error term. The model was fit using means of normal score-transformed values for each transect.

##### 2.2.3.2 Groundwater elevation comparisons

Daily mean, minimum, and maximum tide levels were extracted from water level data sets using package VulnToolkit (Hill and Anisfeld, 2021) in R version 3.5.2 (R Core Team, 2018). Where tidal variations were not detected in marsh groundwater records using VulnToolkit, the timing of low and high tides in the channels was used to identify corresponding high and low tides from the groundwater levels. Mean amplitude ratios (A_r_) were calculated for each well as the ratio of the amplitude of the water fluctuation in the marsh well (*A*_*x*_) to the amplitude of the water fluctuation in the tidal channel (*A*_*s*_) (Jiao and Post, 2019).

(3) $${\text{A}}_{r}=\frac{A_{x}}{A_{s}}$$

Hydraulic conductivity was measured twice for focal wells using a bail down test, and saturated hydraulic conductivity was calculated according to Hvorslev’s Method (Hvorslev, 1951) for an unconfined groundwater aquifer:

(4) $$\text{K}=\frac{r^{2}\ln\frac{L_{e}}{r}}{2L_{e}}\frac{\ln\frac{h_{1}}{h_{2}}}{t_{2}\;-\;t_{1}}$$

where *K* = hydraulic conductivity in cm s^−1^, *r* = radius of well; *L*_*e*_ = the length of the well or piezometer that is screened; *t*_1_, *t*_2_ = time points during refilling; *h*_1_, *h*_2_ = head height during refilling.

Daily mean, minimum, and maximum groundwater elevations at sites were examined to compare effects of treatment (runneled vs. control) and time (pre- vs. post-runneling) effects using two-factor ANOVAs. Year and treatment were factors, and a year by treatment interaction term was produced. We specified an autoregressive error correlation structure to help account for the non-independence of measurements. Following the BACI design, we first evaluated the interaction term. If the interaction term was significant, control vs. runneled differences would be year-specific and year differences would be treatment-specific. If the interaction was not significant, evaluation would depend on whether the main effect for the given factor was significant. For example, if year was determined to be a significant main effect but the interaction was not, then this would indicate that year differences were consistent regardless of whether the data were from a control or an runneled location and were presented that way. Pairwise differences were evaluated using Bonferroni’s adjustment.

##### 2.2.3.3 Salinity comparison between treatments

Salinity was examined along selected transects at each runneled and control area. The effect of year (pre- vs. post-runneling) on salinity was examined using a multi-factor ANOVA with treatment (control vs. runneled) and time as factors, and transect as a nested random effect within treatment. Pairwise comparisons performed with interaction combinations were carried out using Bonferroni adjustment.

## 3 Results

### 3.1 Vegetation transect data

**FIGURE 4:**
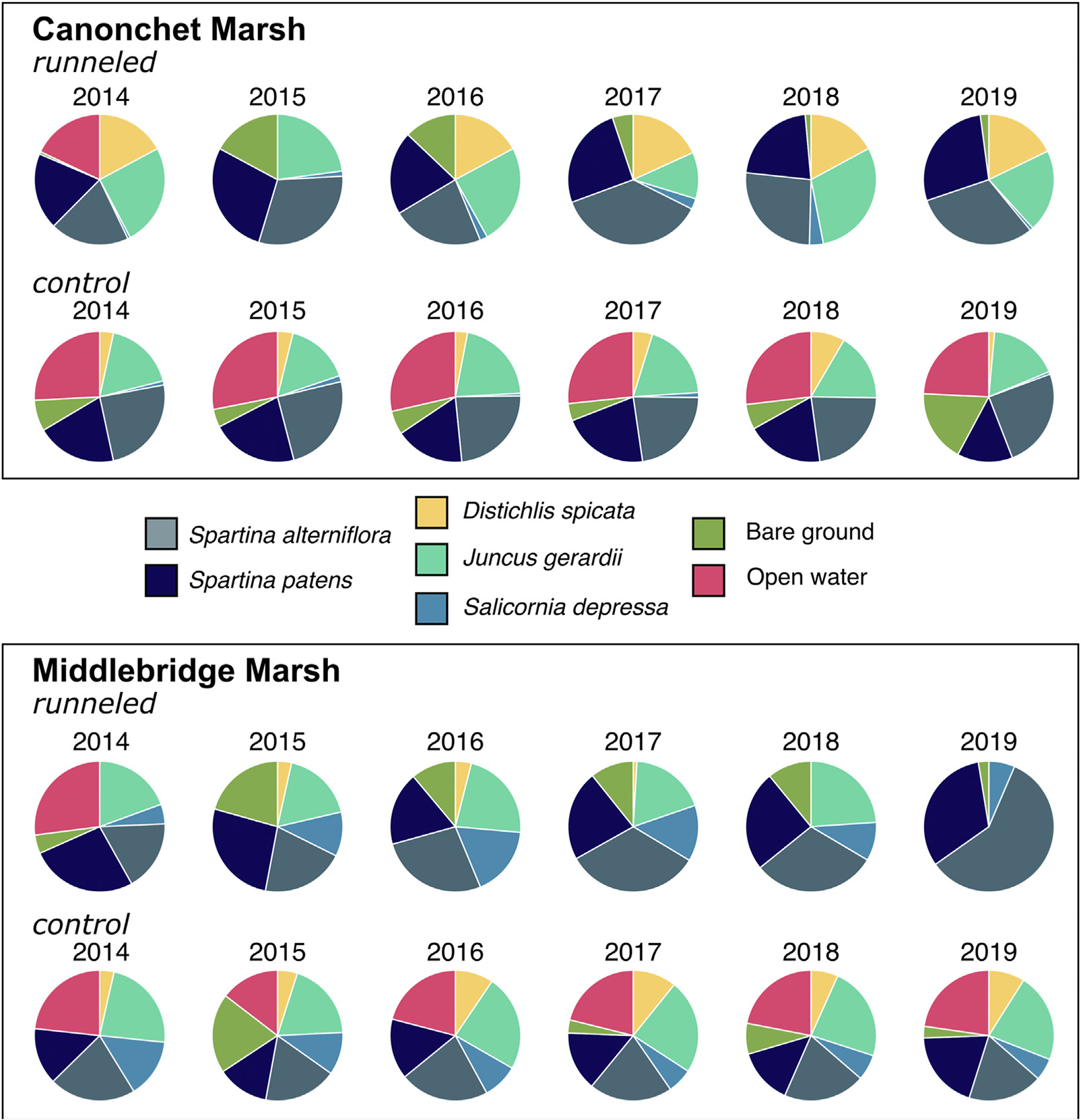
Relative abundance of *S. alterniflora*, *S. patens, D. spicata, J. gerardii, S. depressa,* and area of open water and bare ground along runneled and control transects at Canonchet and Middlebridge in 2014 (pre-runneling) through 2019. Runneling occurred in 2015 and 2016.

At Canonchet, the area of bare ground coverage was greater prior to runnel construction than it was post-runneling (2017–2019) for runneled locations, while at the control locations there was no significant difference between pre- and post-runneling bare ground coverage (Figure 4, Supplementary Figure S2; Supplementary Table S1). Before runneling, there was no difference between control and runneled locations, but after runneling, control areas had greater bare ground cover (10.5% in control areas vs. 0.94% in runneled areas in 2019). Similar to Canonchet, at Middlebridge there was a significant (*p* < 0.001) treatment by time interaction. The pre-runneling bare ground coverage (13.5%) was significantly greater (*p* < 0.001) than the post-runneling bare ground (3.5%) in runneled lobations vs. post-runneling control bare ground (9.0%).

#### 3.1.1 *Spartina alterniflora* coverage

The coverage of *S. alterniflora* significantly increased over time at the runneled locations at Canonchet (*p* = 0.044) (Figure 4, Supplementary Figure S2). While there was no difference in coverage of *S. alterniflora* between the control and runneled locations prior to runnel installation, the coverage of *S. alterniflor*a was significantly greater at the runneled locations post-runneling at Canonchet (28.4% at runneled vs. 24.2% at control) (*p* = 0.026). Prior to runneling, the coverage of *S. alterniflora* at the Middlebridge site was significantly greater at the control locations compared to the runneled locations (12.2% at runneled areas vs. 29.4% at control areas) (*p* < 0.001), but coverage was significantly greater at the runneled locations post-runneling (33.5% at runneled areas vs. 25.7% at control areas) (*p* < 0.001) (Supplementary Table S1).

#### 3.1.2 *Spartina patens* coverage

Prior to runneling there was no significant difference in the coverage of *S. patens* between the runneled and control locations at the Canonchet site (11.3%), but post-runneling, the coverage of *S. paten*s was significantly greater at the runneled locations compared with the control locations (14.9% at runneled areas vs. 8.1% at control areas) (*p* = 0.001) (Figure 4, Supplementary Figure S2; Supplementary Table S1). In contrast, the coverage of *S. patens* at the Middlebridge site was significantly greater (*p* = 0.008) at the control locations (12.2%) compared with the runneled locations (7.6%) post-runneling, and there were no significant differences in coverage pre-runneling (10.6%), although there was a trend towards greater *S. patens* coverage in the control area (Supplementary Table S1).

#### 3.1.3 Plant diversity

At Canonchet, species richness increased at runneled but not control areas (*p* < 0.001; post-runneling > pre-runneling). The treatment by time interaction for species richness was significant (*p* = 0.020) at the Middlebridge site. Prior to runneling the control locations at Middlebridge had significantly greater (*p* = 0.006) species richness, but post runneling there was no significant difference between the runneled and control treatments. At Canonchet, the SDI increased over time in runneled but not control areas (Supplementary Table S1). At Middlebridge, the SDI was significantly greater (*p* < 0.001) at control than runneled locations.

### 3.2 Satellite imagery analysis

**FIGURE 5:**
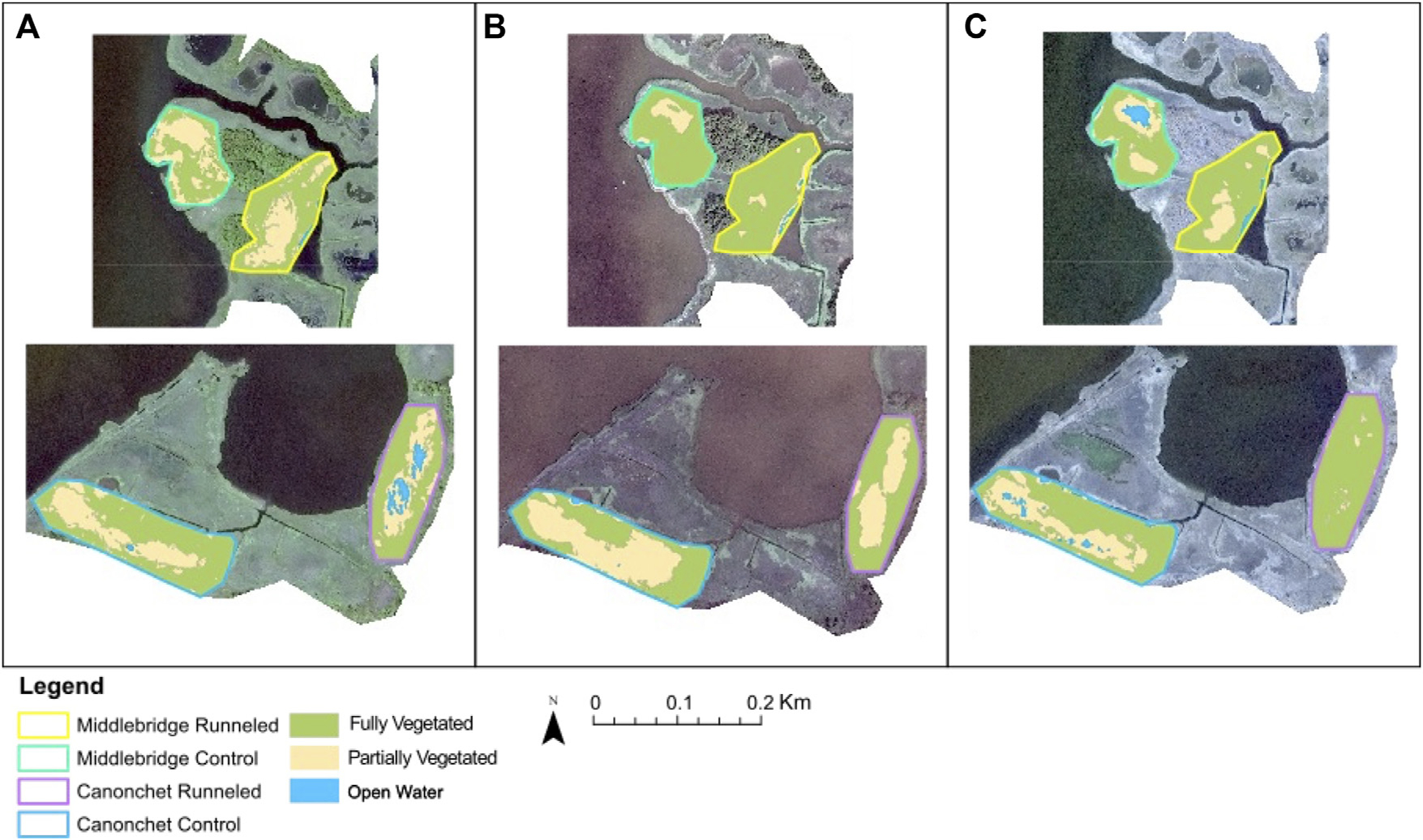
Satellite imagery analysis showing **(A)** 2014; **(B)** 2016; **(C)** 2019. The top row shows Middlebridge and the bottom shows Canonchet.

Analysis of satellite imagery suggested that fully vegetated habitat cover increased in both runneled locations, while mixed results were found in the control areas (Figure 5; Supplementary Table S2). At Canonchet, the fully vegetated area within the runneled site increased from 60.4% to 96.7% between 2014 and 2019, while in the control area the fully vegetated area decreased from 67.8% to 61.5%. Trends in open water and patchy vegetation (<25% plant cover) cover were opposite, where patchy vegetation coverage decreased from 30.8% to 3.3% in the runneled area at Canonchet, while patchy vegetation cover increased from 31.8% in 2014 to 34.9% in the control area. At Middlebridge, the area that was fully vegetated in the runneled area increased from 47.5% to 74.5% between 2014 and 2019, while in the control area, the fully vegetated area increased in extent from 52.1% to 63.8%. While the control area at Middlebridge was found to increase in vegetation extent, the increase in full vegetation coverage was 2.3 times greater in the runneled area than the control area. Patchy vegetation coverage at the runneled site at Middlebridge decreased from 54.4% to 25.5%, while at the control site patchy vegetation decreased from 47.9% to 27.9%. Band index calculations suggested a decrease over time in open water and bare soil in the runneled but not control area at Canonchet, but no difference was found at Canonchet (Supplementary Figure S2).

### 3.3 Water table

**FIGURE 6:**
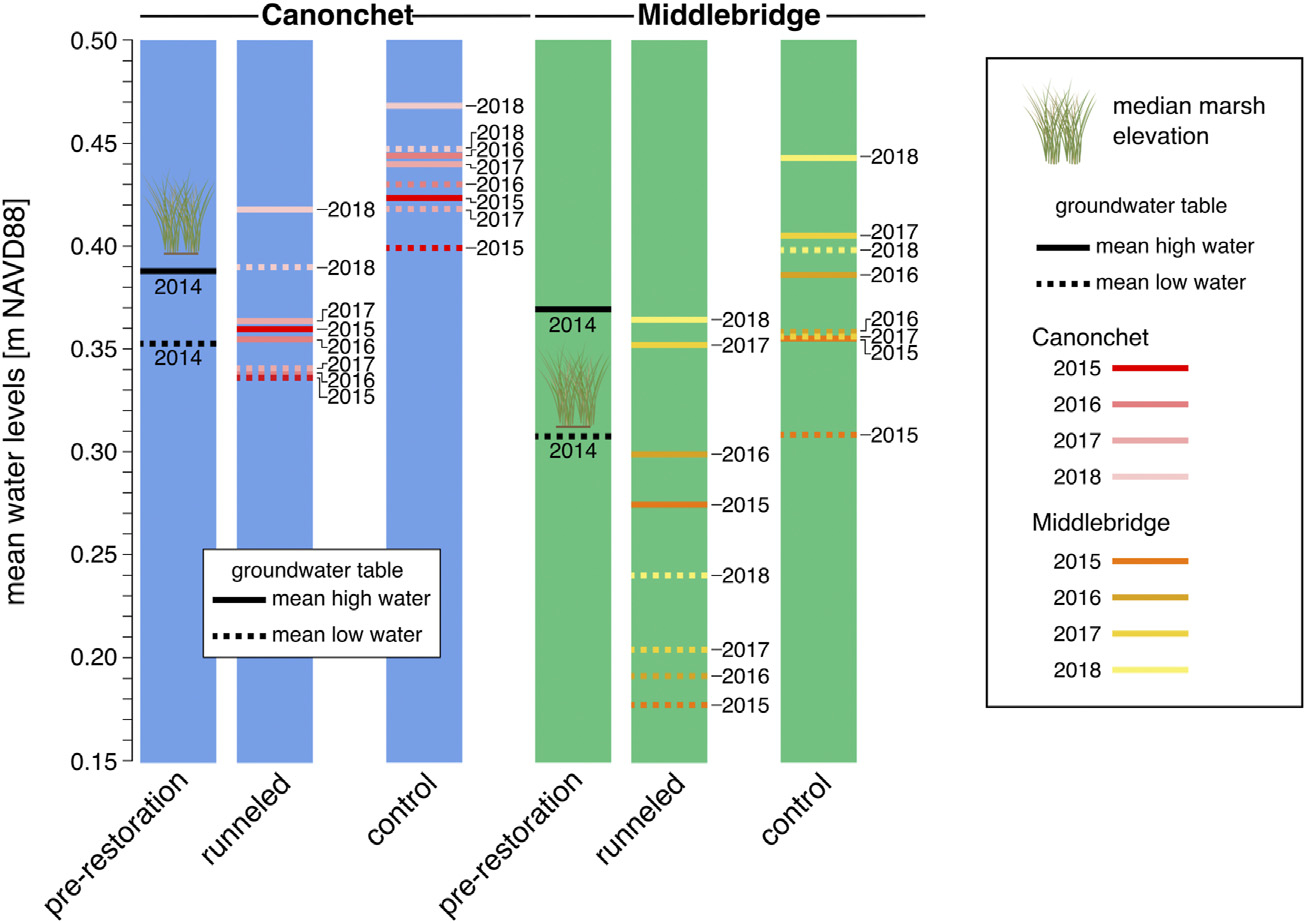
Water levels at Canonchet and Middlebridge. Mean marsh elevation is denoted on the figure using a grass icon. Overall, water levels were lower in runneled areas; at Middlebridge tidal range was also greater in runneled areas. Mean, standard deviation, and number of observations can be found in Supplementary Table S6.

**FIGURE 7:**
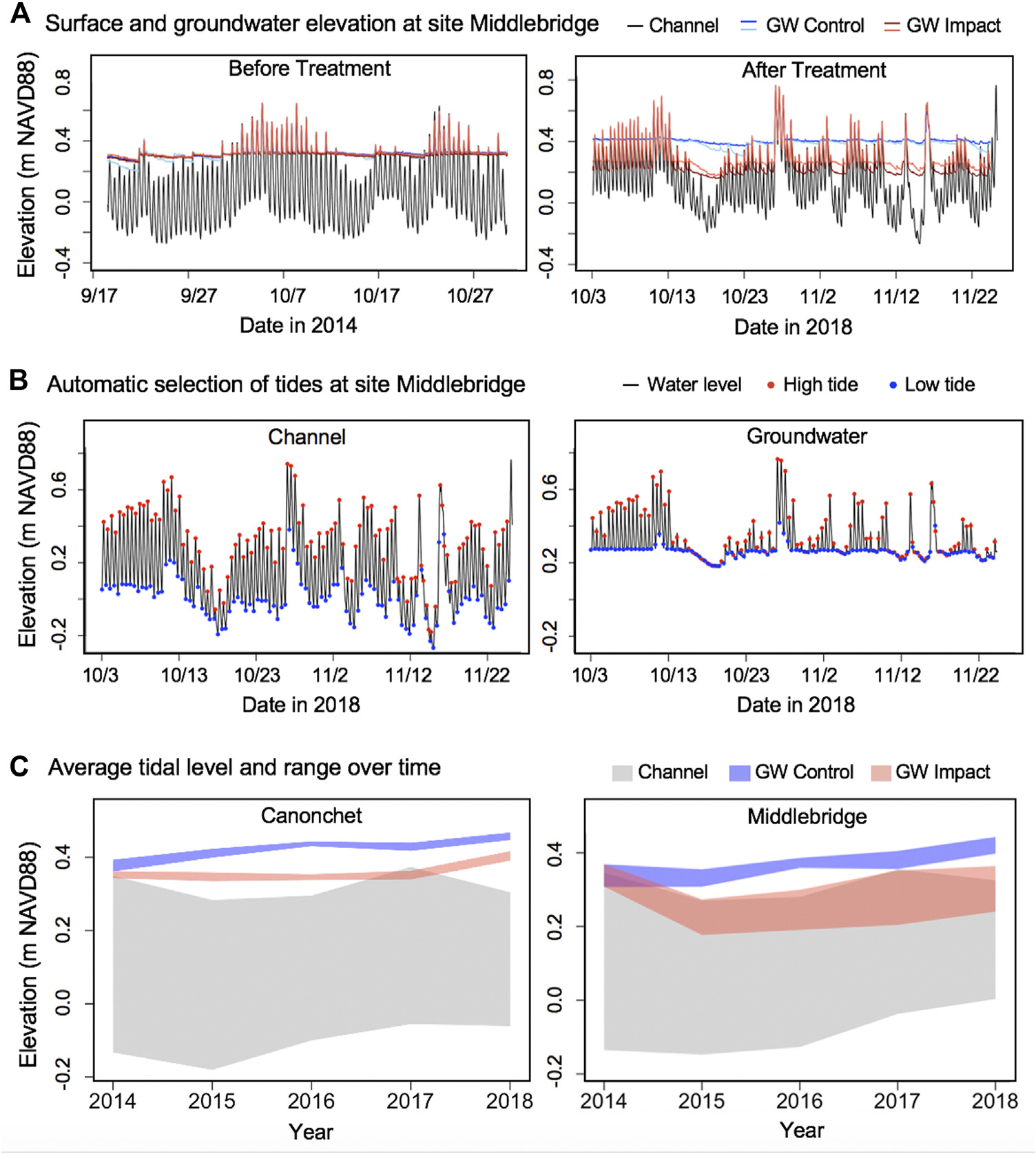
Groundwater levels before and after runnel treatment including: **(A)** Channel surface water and groundwater elevation at groundwater wells at Middlebridge marsh in the first and final year; **(B)** Example result of automatic selection of the high and low tides in the channel and the groundwater datasets using the VulnToolkit package; **(C)** Tidal level and range in the channel and the groundwater wells in the control versus runneled (or impact) treatments. The shaded ribbon represents the tidal range from the average high tide level to the average low tide level each year. The high and low tide levels of the two wells in the control treatment and the two wells in the runneled treatment were each averaged together. Surface and groundwater elevation and automatic selection of tides at Canonchet can be found in Supplementary Figure S5.

The mean groundwater table elevation at Canonchet was 0.36 m NAVD88 in control and runneled areas in 2014 (Figures 6, 7). In 2015, the water levels averaged 0.40 m NAVD88 in control areas and 0.34 m NAVD88 in runneled areas; water levels on average were 6-cm lower in the runneled area. From 2015 to 2018, water levels at Canonchet averaged 7-cm lower in the runneled vs. control areas. The mean groundwater table elevation at Middlebridge was 0.32 m NAVD88 in control and runneled areas in 2014 (Figures 6, 7). Starting in 2015, the runneled areas had water table elevations that were on average 12-cm lower in the runneled vs. control areas. Overall, there were positive upward trends in water table elevations of 1.7–1.9 cm yr^−1^ for the control areas, and the runneled areas excluding the 2014–2015 period (during which a drop in water levels was observed).

Mean amplitude ratios were 0.061 at Canonchet in 2014, meaning that the tidal range in the groundwater table was 6.1% what was observed in the tidal channel. This mean amplitude ratio averaged 0.039 in 2015 through 2018. There was no observable difference in the mean amplitude ratio between control and runneled areas at Canonchet. In contrast, there were observed differences at Middlebridge. The mean amplitude ratio was 0.106 in 2014, and the ratio averaged 0.273 in runneled areas 2015–2018, and 0.092 in the control areas, meaning the runneled area had an 18% greater amount of tidal exchange.

Hydraulic conductivity was found to vary more across Canonchet than Middlebridge, with values as high as 0.3 cm s^−1^ at two locations and as low as 0.002 cm s^−1^. Values were higher at Canonchet than at Middlebridge, although there was heterogeneity observed within sites as well (Supplementary Figure S4; Supplementary Table S3).

Groundwater daily maximum elevation was significantly greater (*p* = 0.0117) for control than runneled locations at Canonchet but not Middlebridge (Supplementary Table S4). The main effects of treatment and time were not significantly different for groundwater daily minimum or daily mean elevation, but there were significant treatment by time interactions. Groundwater elevations were generally greater for control than runneled sites in later years at Canonchet, but no statistical differences were evident at Middlebridge (Supplementary Table S4).

### 3.4 Porewater salinity

There was a significant (*p* = 0.020) treatment by time effect on salinity at Canonchet (Supplementary Table S5). At both the runneled and control locations there were greater salinities post-runneling (27.7 ± 10.3) compared with pre-runneling (20.5 ± 10.5) (mean ± SD). Salinity was similar at the runneled and control locations pre-and post-runneling (29.3 ± 12.3 in runneled areas vs. 26.7 ± 9.8 in control areas). In contrast at Middlebridge, while there was no effect of time on the salinity at the runneled and control locations, the magnitude of the salinity at the control locations was significantly greater than the runneled locations prior to (33.1 ± 5.5 vs. 24.8 ± 8.7) and after (35.8 ± 6.4 vs. 25.0 ± 8.1) runneling.

## 4 Discussion

### 4.1 Runnels as a tool to build ecosystem resilience

The installation of channel extension features, or runnels, in Northeastern US marshes is an approach adapted from mosquito management techniques used in Australia to reduce mosquito populations (Dale, 2008). Runnel installation in the US Northeast has involved excavating shallow drainage features with the aim of increasing the drainage of surface water impounded by topographic highs (both natural and human made features) to allow for recolonization of vegetation, but avoiding the negative impacts that have been observed from mosquito ditching (e.g., peat oxidation, erosion, tidal water impoundment, and subsidence) (Dale and Hulsman, 1990). Runnels are different from the pervasive mosquito ditches installed in Northeastern marshes (Kennish, 2001). They tend to be very shallow, so as to prevent root oxidation (Besterman et al., 2022); although it is recognized that mosquito ditches often started out narrow and shallow as well (Penny, 2010). They are simple tidal channel extension features (Taylor et al., 2020) and work with the natural hydrology. Mosquito ditches, in contrast, often had a high channel density, were installed as linear or gridded features, and often supplanted the pre-existing tidal channel hydrology. Over the past decade, runnels have been introduced in several sites in Rhode Island, at Buzzards Bay, Massachusetts, and at Cape May National Wildlife Refuge in New Jersey (Wigand et al., 2017; Weis et al., 2021). While the Australian literature suggests runnels are a successful mosquito control technique associated with minimal marsh damage (Dale and Knight, 2006), there has not previously been strong quantitative data available to demonstrate their ability to promote positive ecosystem benefits nor effects on the marsh water table in the Northeastern US. Rather, previous work has reported somewhat ambiguous effects (Raposa et al., 2019; Besterman et al., 2022) and with lower carbon dioxide assimilation at runneled locations than for reference sites, suggesting that runnels did not fully recover ecosystem function (Perry et al., 2022).

The formation of marsh ponds and associated vegetation die-off and habitat fragmentation is a principal mode of marsh loss in the Northeastern US (Figure 1) (Watson et al., 2017), and because constructing runnels is a relatively inexpensive and low disturbance intervention, runnel installation could be successfully employed at a wide number of locations. In the present study, we focused on analyzing effects of runneling on marsh vegetation, groundwater levels, flooding, and porewater salinity. Overall, we found that the area of bare ground decreased in runneled but not control areas, and *S. alterniflora* increased at runneled but not control sites, but differences in the cover of other plant species were site specific (Figure 4, Supplementary Figure S2; Supplementary Table S1). Overall, runnels did not consistently increase coverage of high marsh species *Juncus gerardii* or *S. patens* in this or a related study (Besterman et al., 2022). These species are restoration targets as they comprise nesting habitat for the saltmarsh sparrow (*Ammodramus caudacutus*) that is threatened with extinction (Elphick et al., 2015). Although anecdotal reports suggest use of drained areas by marsh-breeding birds (Besterman et al., 2022), it is unclear if runneling improves nesting habitat.

Prior to runnels being installed, the groundwater table at instrumented wells typically sat very near the marsh surface, increased when spring tides flooded the marsh, and decreased during neap tidal cycles when surface flooding did not replenish water lost through evapotranspiration (Figures 2, 7), which has been estimated at 3–6 mm d^−1^ for species of *Spartina, Distichlis,* and *Salicornia* (Moffett et al., 2012). The perched water table found in these marshes pre-intervention can be described as tidal overheight, or the maintenance of the groundwater table in an unconfined coastal aquifer at an elevation significantly above sea level due to increased aquifer transmissivity at high tide (Jiao and Post, 2019). Tidal overheight has been well studied in intertidal habitats, such as beaches and intertidal marsh (e.g., Turner et al., 1997; Xin et al., 2022), and the groundwater table in coastal areas can typically be expected to sit above mean sea level by 20%–25% of the tidal amplitude (Phillip, 1973), although topography and hydraulic conductivity can affect the magnitude of the overheight (Li and Jiao, 2003). This tidal overheight is substantially less adjacent to tidal channels (Figure 2; Xin et al., 2013). The pre-intervention water table condition can help explain why marsh fragmentation and loss is occurring on the marsh platform away from tidal channels, where a perched water table is associated with waterlogged conditions and plant loss. This is a crucial point as high elevation marsh is typically not considered vulnerable to SLR (Cahoon et al., 2019). Although it is ultimately topographic highs that block the exit of surface water, contributing to waterlogging and creating these unvegetated interior depressions, our results suggest that an increase in the groundwater table resulting from SLR can contribute to die-off for high elevation marsh, as the water table intersects and rises above the marsh surface.

We observed increases in the groundwater table at both runneled and unmanipulated control sites over time (Figures 6, 7). In control sites, and in the runneled sites after 2015, the high tide and low tide water table levels increased by an average of 1.7–1.9 cm yr^−1^ from 2014 to 2018. This matched the upward trend in monthly mean high water (MHW) observed at the Newport, RI tide gauge from 2014 to 2018 (1.6-cm yr^−1^; NOAA, 2022b). This short-term increase in mean high water from 2014 to 2018 is both a function of long-term trends and shorter-term variability related to astronomical variables and interannual variability in water levels. However, the rate of rise in monthly MHW over the past 19 years at the Newport, RI tide gauge has been 0.69-cm yr^−1^ (NOAA, 2022b), which is significantly greater than the long-term SLR trend of 0.28-cm yr^−1^ (NOAA, 2022a). While our groundwater table data were not collected consistently during the same months each year, nor do they have as rigorous an elevation control as NOAA tide stations, it does suggest that salt marsh groundwater tables may be rising at a rate that exceeds that of mean sea level, and more closely approximates MHW. Increases in MHW have been observed at rates approaching 1-cm yr^−1^ over the past 19 years across the US Northeast (Courtney et al., 2020; Haaf et al., 2022).

After runnel installation, groundwater levels initially dropped in runneled locations, although they continued to increase over time at similar rates as found in unmanipulated controls (Figure 6). Mean water levels were 7-cm lower in runneled than unmanipulated controls at Canonchet, and 12-cm lower at Middlebridge. Additional differences were observed between the two sites. At Canonchet, there were no differences in the mean amplitude ratio (the fraction of the tidal range in the tidal channel that was transmitted to the marsh groundwater table) before and after runneling. However, at Middlebridge, there was an 18% increase in the in-marsh tidal range at runneled areas. This suggests that installing these small tidal channels can establish marsh hydrology similar to that seen channelside at unrunneled locations where the low-tide water table dips down towards mean sea level (e.g., Figure 2; Xin et al., 2013; Wilson et al., 2015). Because this enhanced drainage occurred in the lower elevation and more frequently inundated marsh, which also had a lower saturated hydraulic conductivity, the explanation for this difference is not straightforward. It may be that the runnels were of slightly deeper depth at Middlebridge, or had a shallowing feature at Canonchet. This deeper depth could have led to greater hydraulic gradients, more overall drainage, and depressed low tide water levels in comparison with more shallowly dug runnels at Canonchet. In fact, Besterman et al. (2022) encouraged the use of “vegetated sills” in runnels to allow for adjustments in drainage after observing effects on the landscape.

Overall, our results suggest that installation of shallow runnels in high elevation infrequently flooded marsh with heterogenous hydraulic conductivity and microtidal conditions (0.43 m daily range of tide) promoted revegetation, as measured through analysis of vegetation transects and satellite imagery analysis, although runnel installation did not clearly promote recolonization of high marsh plant species. This restoration experiment helped establish the role of a rising groundwater table in contributing to upper marsh die-off, by suggesting that the rate of rise in the water table mirrored that of MHW, which has been increasing at rates approaching 1-cm yr^−1^ in the US Northeast. This study also suggests that remediating impacts may be possible with the strategic use of surface water drainage.

### 4.2 Recommendations for future projects

Results from this project can help suggest guidelines that may be used to improve future implementation and monitoring of runnel projects (Table 3). We review suggestions that should improve runnel implementation projects, such as inventorying site characteristics prior to deployment, and designing monitoring campaigns.

Our results suggest that this technique worked better at the higher elevation location (Canonchet), where a minor drop in the groundwater table was sufficient to allow for near-complete vegetation recolonization. On the other hand, the bottom of ponded areas that are too low in elevation to support vegetation should not be expected to recolonize (Besterman et al., 2022). The challenge is how to delineate what “too low” might mean in the US Northeast where tidal range can vary from a few cm to several meters and marsh elevations can vary by over a meter (Elsey-Quirk et al., 2022). A simple and promising indicator may be if the ponded area is recently formed (assessed using historic imagery), shallow, or somewhat ephemeral and the marsh platform has not subsided. A more rigorous assessment could involve elevation surveys or a GIS analysis using LiDAR, if the LiDAR accurately depicts pond elevations (Millette et al., 2010). If the elevation of the pond bed is below the limit for vegetation elsewhere in the marsh, surface drainage will not allow revegetation to occur in the ponded area even if surface water is drained (Mariotti, 2020), yet it can prevent further degradation of marsh vegetation surrounding the ponded area known as “pool creep.”

A second suggestion is to consider additional factors associated with drainage, such as tidal range, and the hydraulic conductivity of the soil. Where tidal ranges are extremely low, gradients are similarly low and runnels may not enhance drainage to the extent that vegetation can recover significantly. In our site, the mean tidal range was ca. 0.40 m, and the water table dropped 0.07–0.12 m based on runnel installation to 0.30 m depth. To operationalize the investigation of tidal range at a candidate site, VDATUM can be used to estimate tidal range in US Northeastern marshes (Haaf et al., 2022); although data is not always available or accurate for back-barrier marshes (Cole Ekberg et al., 2017). In these cases, deployment of an in-channel water level logger for a short time period (e.g., 1–2 months) with data post-processed in R (R Core Team, 2018) using the package VulnToolkit (Hill and Anisfeld, 2021) could help establish tidal range, and can be adjusted using nearby NOAA tide gauge data (NOAA, 2003). Another factor related to tidal range that can help shape drainage in concert with runneling is hydraulic conductivity. At the PRE, we measured saturated hydraulic conductivity that ranged from 10^−5^ to 1 cm s^−1^ and values were quite heterogenous (Supplementary Figure S4). If a marsh has homogeneously fine soils with low hydraulic conductivity, these soils can act as a barrier to through-marsh drainage. Conversely, soils that are very sandy and permeable can help augment runneling to decrease water logging across the landscape. Hydraulic conductivity can be estimated using bail down tests, or through laboratory soil tests using the falling head method (Hvorslev, 1951; Hwang et al., 2017). Sandy soils may also be more conducive for plant recolonization, as they are better drained (Bradley and Morris, 1990).

An additional recommendation of items to consider in planning runneling projects is SLR rates, or how much time you are buying by installing runnels. In this study, we dropped groundwater levels by 7–12 cm in comparison with control sites. However, given that groundwater levels rose 1.7–1.9 cm yr^−1^ from 2014 to 2018, water levels in runneled sites were up to pre-runneled levels by 2018. Given the longer-term rate of MHW rise of 0.5–1 cm yr^−1^ in the US Northeast, dropping the water table by ca. 10 cm will buy 10–20 years of extra time. In this case, the time bought was only 5 years due to the exceptionally fast rate of rise in water levels 2014–2018. Another consideration is that if runnels are clogged, this could also negate the time “bought” by installing runnels. If runnels fill in with sediment or peat, they may need to be cleared to maintain drained conditions. Managers that been installing and maintaining runnels recommend that they be maintained by hand every 3 years.

The results of the present study can also inform monitoring campaign design. Pre-restoration monitoring was key in establishing impacts of runnels, and although we designed this study using a BACI design, an additional year of pre-intervention monitoring data would have been an even more helpful baseline given interannual variability in vegetation and water levels (Figure 5, Supplementary Figure S2). Our monitoring wells were established prior to knowledge of where runnels would be; in retrospect it would have been more helpful to have located wells in restoration areas that had identical proximities to tidal channel distance to help address co-variability in water levels and landscape position (Montalto et al., 2006; Wilson et al., 2015). We found that groundwater levels changed immediately after runnel installation; while vegetation changed more slowly (Figure 5, Supplementary Figure S2). Vegetation transects were helpful for monitoring changes in species cover (Figure 4, Supplementary Figure S2); satellite imagery posed more problems due to differences in tidal levels and season (Morgan et al., 2022). Ideally, drone photographic mosaics could have been used to track change over time (Haskins et al., 2021); however, policies related to the use of drones on USFWS property discouraged their use (50 CFR 27.34, 50 CFR 27.51). Finally, a previous study suggested that enhanced drainage may lead to loss of elevation (Raposa et al., 2019), perhaps due to consolidation and dewatering. While the creeks examined in that study were much deeper and wider than the small channel extension features that we focused on, the potential linkage between channel installation and subsidence is worthy of additional study (Table 3).

## 5 Conclusion

Our results suggest that an increase in the groundwater table resulting from SLR can contribute to vegetation die-off for high elevation marsh, as the water table intersects and rises above the marsh surface. Runnels, or the installation of channel extension features, can help mitigate this adverse effect of the water table rising and pond formation with subsequent die-off. While we acknowledge that runnels may be a temporary solution, we also found that they are also quick acting, with drops in groundwater appearing as soon as the runnels were installed, and vegetation reestablishment occurring in two to 3 years. Runnels also might be a more feasible climate change adaptation technique where sediment addition is not possible, due to cost, distance from sediment sources, or concern about disturbance. We also propose that runnels—even if they do not fully reestablish vegetation—may be helpful in reducing the amount that ponds might expand due to wind-wave erosion or excessive waterlogging. In addition, runnels may promote reductions in the presence of marsh-breeding mosquitos. We suggest that future studies include strong monitoring to guide implementation, and recommend this technique as one of the many tools that are needed to address the effects of climate change on coastal areas over the next centuries.

## Supplementary Material

Supplement1

Supplement2

## Figures and Tables

**TABLE 1:** Dates of vegetation monitoring and satellite image collection used for vegetation change analysis. Tide level is from the Newport, RI, tide station. All satellite images were 4-band, pansharpened imagery with 0.5 m spatial resolution.

Vegetation monitoring	Satellite imagery date	Satellite	Tide level (m)

18 September 2014	5 July 2014	World View-2	0.76
5 August 2015	2 August 2015	World View-2	−0.09
7 August 2016	29 August 2016	World View-2	0.76
12 September 2017	12 June 2017	World View-3	0.09
21 August 2018	23 March 2018	World View-3	0.30
16 September 2019	4 April 2019	World View-2	0.15

**TABLE 2:** Dates when wells were instrumented with water level loggers. Four wells and two channels were instrumented, and an air pressure logger was deployed at Middlebridge in a shaded upland area. Figure 3 depicts the location of instrumented wells and loggers.

Dates	Loggers	Purpose

18 September-30 October 2014	Solinst levelogger 5	Well monitoring
9 October-18 November 2015	Hobo U20L	Well monitoring
22 October-14 December 2016	Hobo U20L	Well monitoring
22 September-27 November 2017	Hobo U20L	Well monitoring
2 October-26 November 2018	Hobo U20L	Well monitoring
14 September 2020	Hobo U20L	Bail down test
7 October 2020	Hobo U20L	Bail down test

**TABLE 3:** Assessment indicators and runneling recommendations for restoration projects.

Indicator	Goal	Methodology	Recommendation
Pond hypsometry	Compare pond bed elevations with elevations that can support vegetation elsewhere	Digital elevation model or measurements using LiDAR or elevation surveys	If pond beds are low in the tidal frame, plants will not be able to recolonize regardless of drainage. Runneling may prevent the pond from expanding. Shallow areas may revegetate
Tidal range	Assess tidal range to estimate extent of possible plant recolonization	Measure tidal range using VDATUM or deployment of in-channel loggers in a large, deep tidal channel or embayment	Very low tidal range sites have less capacity to drain. Drainage enhancements in sites with a <0.20 m tidal range may not realize improvements
Landforms and historic data	Assess past human impacts and modifications to hydrology	Historic areal imagery and ground assessments of drainage features and disturbances (e.g., stone walls, embankments, fill)	Enhance drainage using existing features. Historic imagery and surveys can reveal the origin and cause of impounded water areas
Hydraulic conductivity	To determine whether soil characteristics will enhance or obstruct drainage	Installation of shallow wells, bail down-tests. Collection of soils; measurement in lab (e.g., KSAT, Meter instruments)	Soils with high hydraulic conductivity will enhance drainage; if soils have homogeneously low hydraulic conductivity, drainage improvements may not occur
Sea level rise rate	Determine the amount of time bought by installing runnels. Consider future landscapes and marsh migration pathways	Examine mean monthly trends in MHW registered at nearest local tide gauge for the past 19 years against anticipated changes in soil flooding. Examine DEM, LiDAR, or SLAM maps	If trends in MHW are high, runnels will be a temporary solution. Consider opportunities for restoring tidal hydrology to facilitate marsh migration
Subsidence	Determine whether subsidence or peat oxidation is linked to drainage	Collect baseline elevations using appropriate survey methods (e.g., RTK or PPK GPS, or leveling to a stable upland benchmark)	This technique may not be appropriate if it is linked to significant elevation loss, and it is unknown based on this study

## Data Availability

All data is available as Supplementary Material. Inquiries can be directed to the corresponding author.
