# Supplementary material for: Runnels mitigate marsh drowning in microtidal salt marshes
Source: Front Environ Sci. Author manuscript; Available in PMC 2023 Nov 3. (PMC9728634; doi:10.3389/fenvs.2022.987246)
Supplement: Supplement1 [file NIHMS1848987-supplement-Supplement1.docx]

Supplementary Material

# Supplementary Data

All data was uploaded as supplementary material.

# Supplementary Figures and Tables

## Figures


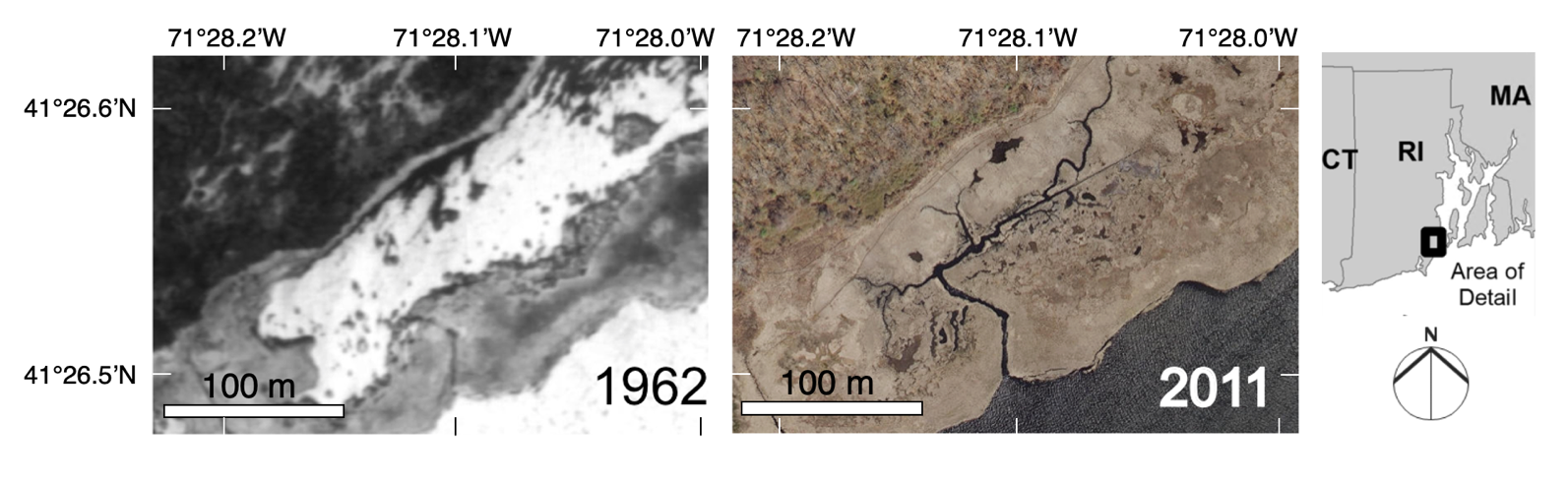


**Supplementary Figure 1.** Pond capture by tidal channel network at the Narrow River Estuary. Tidal creek incision drained the pond (shown at left in the 1962 imagery) and allowed vegetation to reestablish (show at right in the 2011 imagery). In 1939 imagery, the pond was a series of small ponds; by 1951, it had coalesced into the large pond shown at left in the 1962 imagery. The pond was captured and drained between 1962 and 1972 and was fully revegetated by 1985, similar to as shown here at right in the 2011 imagery.

**
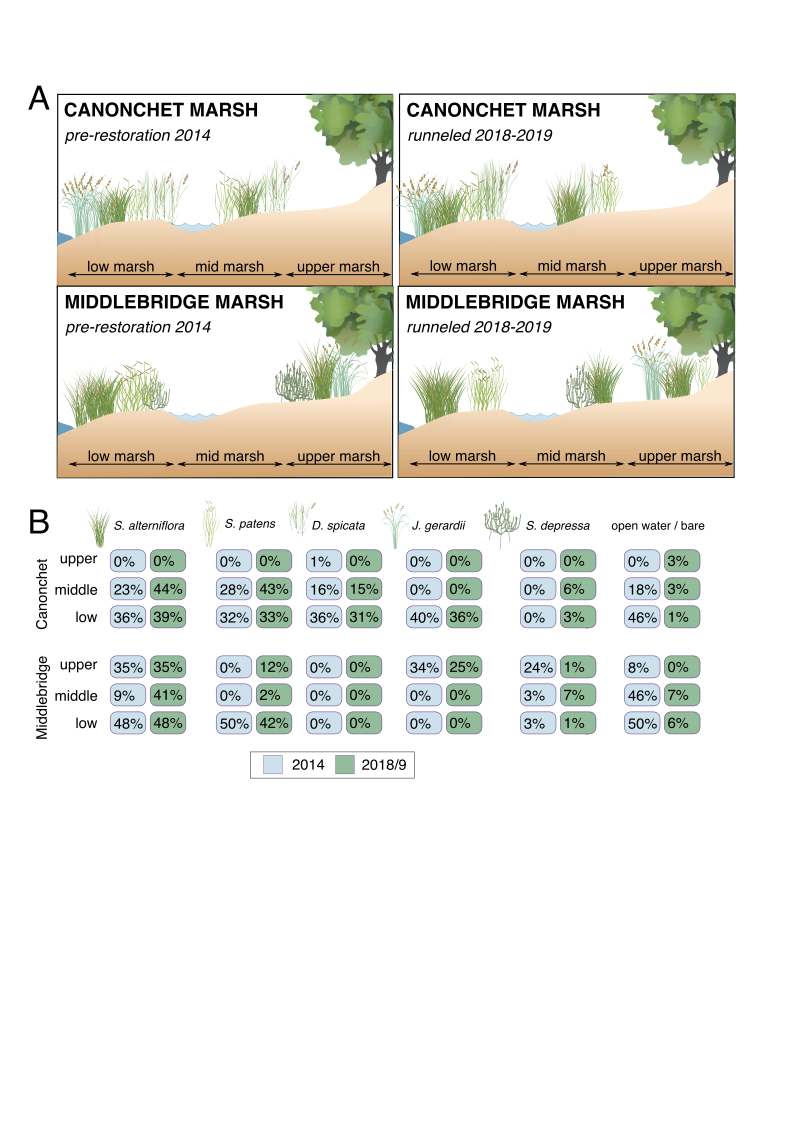
**

**Supplementary Figure 2.** Depiction of the relative abundance of marsh plants at Canonchet and Middlebridge at runneled sites in 2014 and the mean of 2018 and 2019 in the upper marsh (transect plots closest to the upland), mid marsh, and lower marsh (transect plots closest to the estuary). (A) Each plant clump represents 10% cover. (B) blue represents 2014 and green depicts the mean of 2018 and 2019. The upper plots at Canonchet generally support more terrestrial species causing empty area in the figure that lack the marsh plants of interest. Species considered are *Spartina alterniflora, Spartina patens, Distichlis spicata, Juncus gerardii,* and *Salicornia depressa.*


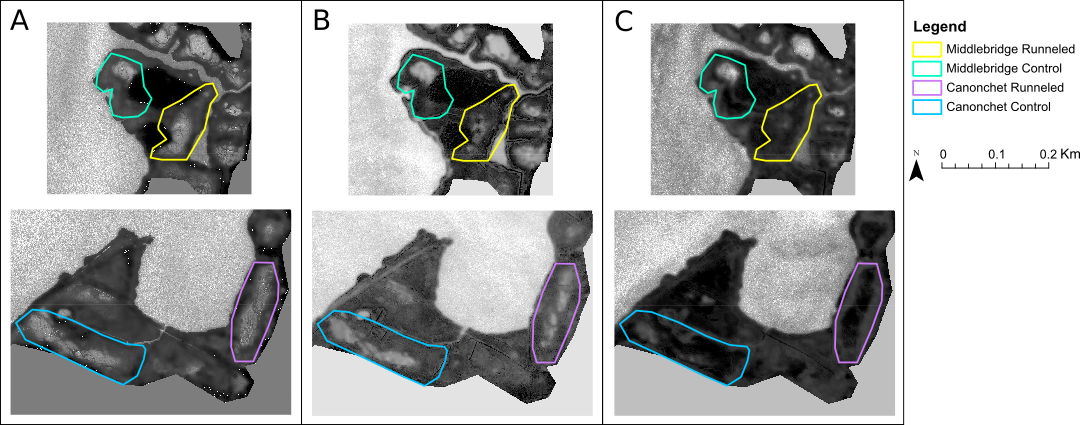


**Supplementary Figure 3.** Satellite imagery band indices at Narrow River study sites depicting the sum of NDWI and BSI showing (A) 2014; (B) 2016; (C) 2019. More reflective values are indicative of open water or bare soil; less reflective or dark values indicate greater vegetation.

*
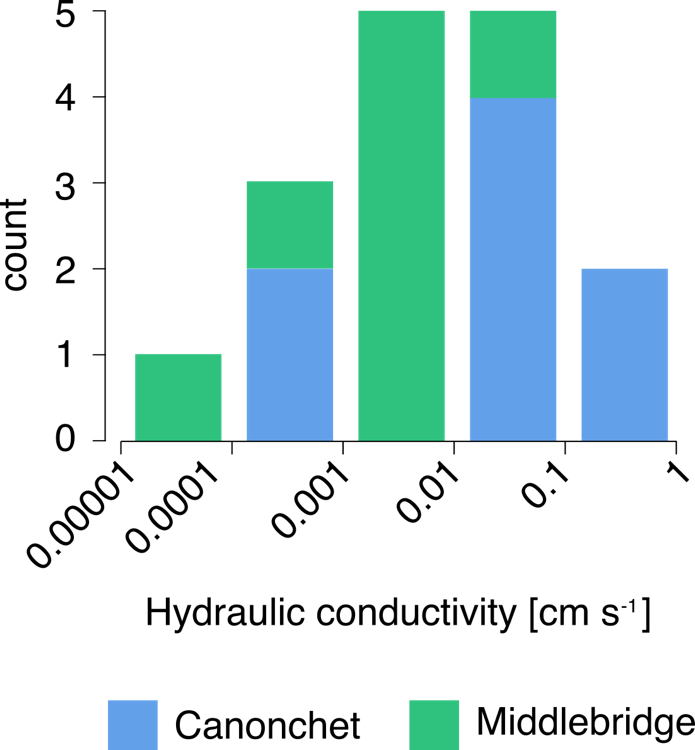
*

**Supplementary Figure 4.** Stacked histograms of measures of hydraulic conductivity made at Canonchet and Middlebridge in the Pettaquamscutt River Estuary. Location and timing of measures can be found in Table 2.


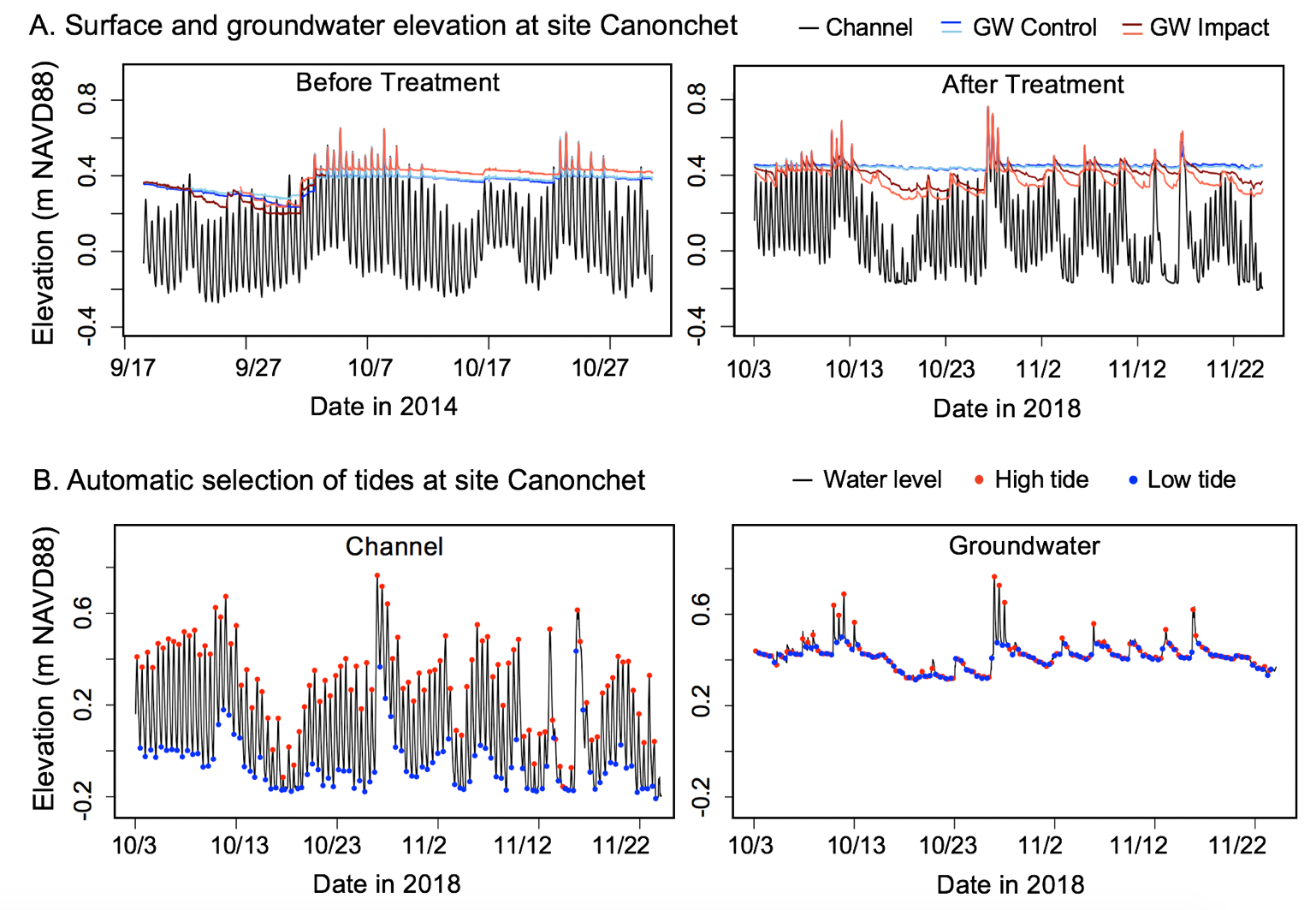


**Supplementary Figure 5.** Groundwater levels before and after runnel treatment including: (A) Channel surface water and groundwater elevation at groundwater wells at Canonchet marsh in the first and final year; (B) Example result of automatic selection of the high and low tides in the channel and the groundwater datasets using the VulnToolkit package.

## Tables

**Supplementary Table 1.**

Results of a omparison of vegetation cover (bare and dominant plant species), species richness, and the Shannon Diversity Index (SDI) were carried out using a repeated measures, multi-factor ANOVA, with transect nested within the treatment variable. The effects of time (before, 2014; during, 2015-2016; after, 2017-2019) and treatment (runneled vs. control) were examined for each site (Canonchet and Middlebridge). Evaluations of treatment significance were performed using transect variability as the associated error term, and evaluations of the time by treatment interaction significance and individual pairwise comparisons within the context of that interaction were done using transect by time variability as the associated error term. The model was fit using means of normal score-transformed values for each transect. Because the time lag for vegetation to recover, we did not incorporate vegetation analysis of the during (2015-2016) time period. *MS_b_* is mean sum of squares error between groups, while *MS_w_* is mean sum of squares error within the group; where *F* = $\frac{{MS}_{B}}{{MS}_{W}}$. There was no *Phramites australis* at Middlebridge, so the data is not available.

| **Site** | **Response variable** | **treatment x time** | **Runneled change over time** | **Control change over time** | **Runneled vs. control [pre]** | **Runneled vs. control [post]** |
| --- | --- | --- | --- | --- | --- | --- |
| Canonchet | bare | *F*_4,18_ = 3.04 *MS_b_* =0.10  *MS_w_* =0.03  *p*=0.0443 | pre>post  *t*  = -2.63  *df* = 18  *p*=0.0169 | *t*  = 1.83  *df* = 18  *p*=0.0843 | *t*  = 0.44  *df* = 18  *p*=0.6671 | control>runneled  *t*  = -5.55  *df* = 18  *p*<0.0001 |
| Middlebridge | bare | *F*_4,14_ = 14.72  *MS_b_* =0.26  *MS_w_* =0.02 *p*<0.0001 | pre>post  *t* = -6.39  *df* = 14  *p*<0.0001 | pre<post  *t*  = 2.77  *df* = 14  *p*=0.0150 | runneled>control  *t*  = 7.88  *df* = 14  *p*<0.0001 | *t*  = 0.15  *df* = 14  *p*=0.8791 |
| Canonchet | *Spartina alterniflora* | *F*_4,18_ = 2.76  *MS_b_* =0.08  *MS_w_* =0.03 *p*=0.0596 | pre<post  *t* = 2.16  *df* = 18  *p*=0.0442 | *t*  = -1.73  *df* = 18  *p*=0.1007 | *t*  = -1.78  *df* = 18  *p*=0.0914 | runneled>control  *t*  = 2.42  *df* = 18  *p*=0.0264 |
| Middlebridge | *Spartina alterniflora* | *F*_4,14_ = 42.67  *MS_b_* =0.62  *MS_w_* =0.01  *p*<0.0001 | pre<post  *t* = 9.59  *df* = 14  *p*<0.0001 | pre>post  *df* = 14  *t*  = -3.53  *p*=0.0033 | control>runneled  *t*  = -7.84  *df* = 14  *p*<0.0001 | runneled>control  *t*  = 5.91  *df* = 14  *p*<0.0001 |
| Canonchet | *Spartina patens* | *F*_4,18_ = 2.62  *MS_b_*=0.10  *MS_w_* =0.04  *p*=0.0692 | *t* = 1.61  *df* = 18  *p*=0.1242 | *t*  = -1.90  *df* = 18  *p*=0.0741 | *t*  = -0.69  *df* = 18  *p*=0.5019 | runneled>control  *t*  = 3.78  *df* = 18  *p*=0.0014 |
| Middlebridge | *Spartina patens* | *F*_4,14_ = 0.9  *MS_b_* = 0.02  *MS_w_* = 0.02  *p*=0.4880 | *t* = 0.46  *df* = 14  *p*=0.6512 | *t*  = -0.23  *df* = 14  *p*=0.8202 | runneled<control  *t*  = -2.39  *df* = 14  *p*=0.0315 | runneled<control  *t*  = -3.12  *df* = 14  *p*=0.0075 |
| Canonchet | *Phragmites australis* | *F*_4,18_ = 0.47  *MS_b_* = 0.01  *MS_w_* = 0.01  *p*=0.7578 | *t* = 1.65  *df* = 18  *p*=0.1157 | *t*  = 0.91  *df* = 18  *p*=0.3755 | *t*  = 1.40  *df* = 18  *p*=0.1782 | runneled>control  *t*  = 3.48  *df* = 18  *p*=0.0027 |
| Middlebridge | *Phragmites australis* | N/A | N/A | N/A | N/A | N/A |
| Canonchet | *Distichlis spicata* | *F*_4,18_ = 3.61  *MS_b_* = 0.32  *MS_w_* = 0.09  *p*=0.0249 | *t* = 1.10  *df* =18  *p*=0.2869 | *t*  = 0.69  *df* =18  *p*=0.4977 | runneled>control  *t*  = 3.45  *df* =18  *p*=0.0029 | *r*unneled>control  *t*  = 6.55  *df* =18  *p*<0.0001 |
| Middlebridge | *Distichlis spicata* | *F*_4,14_ = 0.58  *MS_b_* = 0.02  *MS_w_* = 0.03  *p*=0.6838 | *t* = 0.27  *df* = 14  *p*=0.7906 | *t*  = 0.64  *df* = 14  *p*=0.5339 | *t*  = -1.10  *df* = 14  *p*=0.2882 | control>runneled  *t*  = -2.27  *df* = 14  *p*=0.0399 |
| Canonchet | *Juncus gerardii* | *F*_4,18_ = 0.30  *MS_b_* = 0.01  *MS_w_* = 0.004  *p*=0.8774 | *t* = 0.85  *df* = 18  *p*=0.4040 | *t*  = -0.23  *df* = 18  *p*=0.8211 | *t*  = -1.97  *df* = 18  *p*=0.0639 | *t*  = -1.89  *df* = 18  *p*=0.0755 |
| Middlebridge | *Juncus gerardii* | *F*_4,14_ = 3.85  *MS_b_* = 0.05  *MS_w_* = 0.01  *p*=0.0260 | pre>post  *t* = -3.00  *df* = 14  *p*=0.0096 | *t*  = -1.74  *df* = 14  *p*=0.1044 | *t*  = -1.02  *df* = 14  *p*=0.3261 | control>runneled  *t*  = -1.02  *df* = 14  *p*=0.0006 |
| Canonchet | *Salicornia depressa* | *F*_4,18_ = 1.58  *MS_b_* = 0.20  *MS_w_* = 0.12  *p*=0.2221 | pre<post  *t* = 2.42  *df* = 18  *p*=0.0264 | *t*  = -1.04  *df* = 18  *p*=0.3122 | *t*  = -1.00  *df* = 18  *p*=0.3308 | runneled>control  *t*  = 3.16  *df* = 18  *p*=0.0054 |
| Middlebridge | *Salicornia depressa* | *F*_4,14_ = 10.94  *MS_b_* = 0.49  *MS_w_* = 0.04  *p*=0.0003 | pre<post  *t* = 2.69  *df* = 14  *p*=0.0175 | pre>post  *t*  = -7.03  *df* = 14  *p*<0.0001 | control>runneled  *t*  = -6.21  *df* = 14  *p*<0.0001 | *t*  = 2.11  *df* = 14  *p*=0.0533 |
| Canonchet | Species richness | *F*_4,18_ = 5.57  *MS_b_* = 0.23  *MS_w_* = 0.04  *p*=0.0043 | pre<post  *t* = 4.70  *df* = 18  *p*=0.0002 | *t*  = -1.22  *df* = 18  *p*=0.2389 | *t*  = 0.95  *df* = 18  *p*=0.3535 | runneled>control  *t*  = 10.03  *df* = 18  *p*<0.0001 |
| Middlebridge | Species richness | *F*_4,14_ = 4.14  *MS_b_* = 0.26  *MS_w_* = 0.06  *p*=0.0203 | *t* = 1.61  *df* = 14  *p*=0.1304 | *t*  = -1.35  *df* = 14  *p*=0.1969 | control>runneled  *t*  = -3.24  *df* = 14  *p*=0.0060 | *t*  = -1.41  *df* = 14  *p*=0.1811 |
| Canonchet | SDI | *F*_4,18_ = 2.71  *MS_b_* = 0.20  *MS_w_* = 0.07  *p*=0.0629 | pre<post  *t* = 2.91  *df* = 18  *p*=0.0093 | *t*  = -1.15  *df* = 18  *p*=0.2644 | *t*  = -0.74  *df* = 18  *p*=0.4691 | runneled>control  *t*  = 4.47  *df* = 18  *p*=0.0003 |
| Middlebridge | SDI | *F*_4,14_ = 1.69  *MS_b_* = 0.11  *MS_w_* = 0.06  *p*=0.2085 | pre>post  *t* = -3.79  *df* =14  *p*=0.0020 | pre>post  *t*  = -3.52  *df* =14  *p*=0.0034 | *t*  = -1.87  *df* =14  *p*=0.0827 | control>runneled  *t*  = -4.92  *df* =14  *p*=0.0002 |

**Supplementary Table 2.** Percentage of cover for three categories (patchy vegetation, fully vegetated, open water) for runneled and reference sites at Middlebridge and Canonchet for 2014-2019.

| **Site** | **Treatment** | **Category** | **2014** | **2015** | **2016** | **2017** | **208** | **2019** |
| --- | --- | --- | --- | --- | --- | --- | --- | --- |
| Middlebridge | Runneled | Patchy Vegetation | 52.43 | 63.71 | 5.32 | 51.13 | 53.75 | 25.49 |
| Middlebridge | Reference | Patchy Vegetation | 47.92 | 77.15 | 17.12 | 55.73 | 21.80 | 27.91 |
| Canonchet | Runneled | Patchy Vegetation | 30.82 | 25.53 | 33.84 | 29.51 | 18.47 | 3.29 |
| Canonchet | Reference | Patchy Vegetation | 31.80 | 36.86 | 51.25 | 21.87 | 37.82 | 34.88 |
| Middlebridge | Runneled | Fully vegetated | 47.57 | 33.12 | 94.68 | 48.85 | 46.25 | 74.51 |
| Middlebridge | Reference | Fully vegetated | 52.08 | 22.12 | 82.67 | 35.75 | 69.27 | 63.82 |
| Canonchet | Runneled | Fully vegetated | 60.42 | 74.47 | 66.16 | 70.49 | 81.53 | 96.71 |
| Canonchet | Reference | Fully vegetated | 67.83 | 60.69 | 48.28 | 73.89 | 54.85 | 61.51 |
| Middlebridge | Runneled | Open water | 0.00 | 3.16 | 0.00 | 0.01 | 0.00 | 0.00 |
| Middlebridge | Reference | Open water | 0.00 | 0.73 | 0.21 | 8.51 | 8.93 | 8.27 |
| Canonchet | Runneled | Open water | 8.76 | 0.00 | 0.00 | 0.00 | 0.00 | 0.00 |
| Canonchet | Reference | Open water | 0.37 | 2.46 | 0.47 | 4.24 | 7.33 | 3.61 |

**Supplementary Table 3.** Results of the bail down test used to calculate hydraulic conductivity (*K*), showing the variables input into Hvorslev’s Method equation (Hvorslev 1951)

| Site | Treatment | Well Code | Date | *r*  (cm) | *L_e_*  (cm) | *t*_1_  (s) | *t*_2_  (s) | *h*_1_  (cm) | *h*_2_  (cm) | *K* (cm/s) |
| --- | --- | --- | --- | --- | --- | --- | --- | --- | --- | --- |
| Canonchet | Control | T1.40 | 14 Sept 2020 | 10.2 | 70 | 100 | 200 | 10.1 | 35.5 | 0.0178 |
| Canonchet | Control | T3.20 | 14 Sept 2020 | 10.2 | 70 | 1 | 30 | 25 | 42.3 | 0.0250 |
| Canonchet | Runneled | T3.50 | 14 Sept 2020 | 10.2 | 70 | 10,000 | 20,000 | 1.6 | 4.1 | 0.00014 |
| Canonchet | Runneled | T5.25 | 14 Sept 2020 | 10.2 | 70 | 100 | 1100 | 4.0 | 6.9 | 0.00078 |
| Canonchet | Control | T1.40 | 7 Oct 2020 | 10.2 | 70 | 1 | 5 | 2.1 | 4.5 | 0.268 |
| Canonchet | Control | T3.20 | 7 Oct 2020 | 10.2 | 70 | 10 | 50 | 12.7 | 21.2 | 0.018 |
| Canonchet | Runneled | T3.50 | 7 Oct 2020 | 10.2 | 70 | 10 | 30 | 12.5 | 36.8 | 0.077 |
| Canonchet | Runneled | T5.25 | 7 Oct 2020 | 10.2 | 70 | 1 | 5 | 12.3 | 31.2 | 0.331 |
| Middlebridge | Control | T3.36 | 14 Sept 2020 | 10.2 | 70 | 100 | 600 | 17.0 | 20.7 | 0.00056 |
| Middlebridge | Control | T5.24 | 14 Sept 2020 | 10.2 | 70 | 1 | 6,000 | 15.6 | 20.8 | 0.00007 |
| Middlebridge | Runneled | T1.45 | 14 Sept 2020 | 10.2 | 70 | 200 | 300 | 12.2 | 28.4 | 0.0060 |
| Middlebridge | Runneled | T3.30 | 14 Sept 2020 | 10.2 | 70 | 100 | 400 | 16.8 | 46.3 | 0.0048 |
| Middlebridge | Control | T3.36 | 7 Oct 2020 | 10.2 | 70 | 20 | 60 | 39.4 | 53.1 | 0.011 |
| Middlebridge | Control | T5.24 | 7 Oct 2020 | 10.2 | 70 | 50 | 150 | 17.7 | 20.4 | 0.002 |
| Middlebridge | Runneled | T1.45 | 7 Oct 2020 | 10.2 | 70 | 100 | 200 | 34.8 | 43.1 | 0.003 |
| Middlebridge | Runneled | T3.30 | 7 Oct 2020 | 10.2 | 70 | 100 | 200 | 35.6 | 46.8 | 0.004 |

**Supplementary Table 4.** Daily mean, minimum, and maximum groundwater elevations at sites were examined to compare effects of treatment (runneled vs. control) and time (year) using two-factor General Linear Models. Year and treatment were factors, and a year by treatment interaction term was produced. We specified an autoregressive error correlation structure to help account for the non-independence of measurements, as our water level logger data were collected at 15 minute intervals. Pairwise differences were evaluated using Bonferroni’s adjustment. NI indicates non-interpreted.

| **Site** | **Measure** | **Time x treatment** | **Time main effect** | **Treatment main effect** | **Time differences** | **Treatment differences** |
| --- | --- | --- | --- | --- | --- | --- |
| Canonchet | Daily mean | *F*_4,8_=4.47  *p*=0.0344 | NI | NI | NI | 2014: no difference;  2015: control>runneled (*t*=4.88; *df*=8; *p*=0.0037);  2016: control>runneled (*t*=3.63; *df*=8; *p*=0.0201);  2017: control>runneled (*t*=4.29; *df*=8; *p*=0.0053);  2018: no difference |
| Middlebridge | Daily mean | *F*_4,8_=5.91  *p*=0.0163 | NI | NI | control:2014<2018 (*t*=-4.04, *df =* 8, *p=*0.0075);  runneled: 2014>2017 (*t*=3.66, *df =* 8, *p=*0.0128) | 2014: runneled>control (*t*=-3.96, *df*=8; *p*=0.0125);  2015-2018: no difference |
| Canonchet | Daily minimum | *F*_4,8_=7.04  *p*=0.0098 | NI | NI | control: 2014>2017(*t*=5.41; *df*=8; *p*=0.0013), 2014>2018 (*t*=4.24; *df*=8; *p*=0.0057); runneled: 2014>2017 (*t*=6.18; *df*=8; *p*=0.0005) | 2014: no difference;  2015: control>runneled *t*=3.76; *df*=8; *p*=0.0167);  2016: no difference;  2017: control>runneled (*t*=2.89; *df*=8; *p*=0.0406); |
| Middlebridge | Daily minimum | *F*_4,8_=12.15  *p*=0.0018 | NI | NI | control: 2014<2017 (*t*=-3.96; *df*=8; *p*=0.0084), 2014<2018 (*t*=-7.20; *df*=8; *p*=0.0002); runneled 2014>2017(*t*=3.28; *df*=8; *p*=0.0224) | 2014-2018: no differences |
| Canonchet | Daily maximum | *F*_4,8_=0.55 *p*=0.7076 | *F*_4,8_=3.77 *p*=0.0520 | *F*_1,2_=83.71 *p*=0.0117 | NI | 2014-2018: control>runneled  (based on F-test) |
| Middlebridge | Daily maximum | *F*_4,8_=0.59  *p*=0.6778 | *F*_4,8_=3.28 *p*=0.0720 | *F*_1,2_=10.88 *p*=0.0809 | NI | NI (main effect not sig.) |

**Supplementary Table 5.** Variations in porewater salinity relative to treatment and site were examined using a multi-factor ANOVA. Pairwise comparisons performed with interaction combinations were carried out using Bonferroni adjustment. Contrasts were done for 2017-2018 (late) vs. 2014-2016 (early).

| **Site** | **Treatment x time** | **Runneled change over time** | **Control change over time** | **Runneled vs. Control**  **Early** | **Runneled vs. Control**  **Late** |
| --- | --- | --- | --- | --- | --- |
| Canonchet | *F_4,16_=*5.05  *p* = 0.0199 | late>early  *t* = 4.13  *p* = 0.0008 | late>early  *t* = 2.36  *p* = 0.0310 | no difference  *t* = -1.79  *p* = 0.0931 | no difference  *t* = -0.60  *p* = 0.5601 |
| Middlebridge | *F_4,14_=*0.84  *p* = 0.5235 | no difference  *t* = -1.30  *p* = 0.2143 | no difference  *t* = 0.51  *p* = 0.6162 | control>runneled  *t* = -3.64  *p* = 0.0027 | control>runneled  *t* = -8.98  *p* = 0.0001 |

**Supplementary Table 6.** Mean, standard deviation, and number of observations of high and low tides at Canonchet and Middlebridge instrumented wells and channel loggers.

| **Location** | **Year** | **Tide level** | **location** | **number of tides** | **Mean (m NAVD88)** | **Standard deviation (m)** |
| --- | --- | --- | --- | --- | --- | --- |
| Middlebridge | 2014 | High tide | channel | 81 | 0.35 | 0.14 |
| Middlebridge | 2014 | Low tide | channel | 80 | -0.14 | 0.09 |
| Middlebridge | 2014 | High tide | runneled | 162 | 0.40 | 0.22 |
| Middlebridge | 2014 | Low tide | runneled | 160 | 0.37 | 0.10 |
| Middlebridge | 2014 | High tide | control | 162 | 0.37 | 0.10 |
| Middlebridge | 2014 | Low tide | control | 160 | 0.31 | 0.02 |
| Middlebridge | 2015 | High tide | channel | 73 | 0.27 | 0.17 |
| Middlebridge | 2015 | Low tide | channel | 73 | -0.15 | 0.09 |
| Middlebridge | 2015 | High tide | runneled | 146 | 0.27 | 0.16 |
| Middlebridge | 2015 | Low tide | runneled | 146 | 0.18 | 0.05 |
| Middlebridge | 2015 | High tide | control | 146 | 0.36 | 0.11 |
| Middlebridge | 2015 | Low tide | control | 146 | 0.31 | 0.03 |
| Middlebridge | 2016 | High tide | channel | 98 | 0.28 | 0.16 |
| Middlebridge | 2016 | Low tide | channel | 98 | -0.13 | 0.09 |
| Middlebridge | 2016 | High tide | runneled | 192 | 0.30 | 0.12 |
| Middlebridge | 2016 | Low tide | runneled | 192 | 0.19 | 0.04 |
| Middlebridge | 2016 | High tide | control | 196 | 0.39 | 0.07 |
| Middlebridge | 2016 | Low tide | control | 196 | 0.36 | 0.02 |
| Middlebridge | 2017 | High tide | channel | 127 | 0.36 | 0.15 |
| Middlebridge | 2017 | Low tide | channel | 127 | -0.04 | 0.10 |
| Middlebridge | 2017 | High tide | runneled | 254 | 0.35 | 0.14 |
| Middlebridge | 2017 | Low tide | runneled | 254 | 0.20 | 0.05 |
| Middlebridge | 2017 | High tide | control | 254 | 0.40 | 0.10 |
| Middlebridge | 2017 | Low tide | control | 254 | 0.36 | 0.02 |
| Middlebridge | 2018 | High tide | channel | 106 | 0.33 | 0.19 |
| Middlebridge | 2018 | Low tide | channel | 103 | 0.00 | 0.12 |
| Middlebridge | 2018 | High tide | runneled | 210 | 0.36 | 0.14 |
| Middlebridge | 2018 | Low tide | runneled | 202 | 0.24 | 0.05 |
| Middlebridge | 2018 | High tide | control | 210 | 0.44 | 0.09 |
| Middlebridge | 2018 | Low tide | control | 200 | 0.40 | 0.02 |
| Canonchet | 2014 | High tide | channel | 81 | 0.35 | 0.14 |
| Canonchet | 2014 | Low tide | channel | 80 | -0.13 | 0.09 |
| Canonchet | 2014 | High tide | runneled | 94 | 0.39 | 0.11 |
| Canonchet | 2014 | Low tide | runneled | 94 | 0.36 | 0.08 |
| Canonchet | 2014 | High tide | control | 162 | 0.39 | 0.09 |
| Canonchet | 2014 | Low tide | control | 160 | 0.36 | 0.04 |
| Canonchet | 2015 | High tide | channel | 73 | 0.28 | 0.17 |
| Canonchet | 2015 | Low tide | channel | 73 | -0.18 | 0.08 |
| Canonchet | 2015 | High tide | runneled | 146 | 0.36 | 0.11 |
| Canonchet | 2015 | Low tide | runneled | 146 | 0.33 | 0.05 |
| Canonchet | 2015 | High tide | control | 146 | 0.42 | 0.07 |
| Canonchet | 2015 | Low tide | control | 146 | 0.40 | 0.01 |
| Canonchet | 2016 | High tide | channel | 102 | 0.30 | 0.16 |
| Canonchet | 2016 | Low tide | channel | 102 | -0.10 | 0.03 |
| Canonchet | 2016 | High tide | runneled | 202 | 0.35 | 0.09 |
| Canonchet | 2016 | Low tide | runneled | 200 | 0.34 | 0.06 |
| Canonchet | 2016 | High tide | control | 204 | 0.44 | 0.05 |
| Canonchet | 2016 | Low tide | control | 204 | 0.43 | 0.01 |
| Canonchet | 2017 | High tide | channel | 128 | 0.37 | 0.14 |
| Canonchet | 2017 | Low tide | channel | 127 | -0.06 | 0.09 |
| Canonchet | 2017 | High tide | runneled | 255 | 0.36 | 0.13 |
| Canonchet | 2017 | Low tide | runneled | 254 | 0.34 | 0.09 |
| Canonchet | 2017 | High tide | control | 256 | 0.44 | 0.11 |
| Canonchet | 2017 | Low tide | control | 254 | 0.42 | 0.08 |
| Canonchet | 2018 | High tide | channel | 102 | 0.30 | 0.20 |
| Canonchet | 2018 | Low tide | channel | 100 | -0.06 | 0.11 |
| Canonchet | 2018 | High tide | runneled | 204 | 0.42 | 0.09 |
| Canonchet | 2018 | Low tide | runneled | 200 | 0.39 | 0.05 |
| Canonchet | 2018 | High tide | control | 204 | 0.47 | 0.06 |
| Canonchet | 2018 | Low tide | control | 200 | 0.45 | 0.01 |
