# Supplementary material for: Runnels mitigate marsh drowning in microtidal salt marshes
Source: Front Environ Sci. Author manuscript; Available in PMC 2023 Nov 3. (PMC9728634; doi:10.3389/fenvs.2022.987246)
Supplement: Supplement2 [file NIHMS1848987-supplement-Supplement2.zip › Runnel Data_Submission/0 Metadata Description.docx]

**Experimental Design Overview:**

- Before-After Control-Impact (BACI) study design.
- The “Impact” treatment was installation of runnels in the marsh in 2015 and 2016.
- Two locations (Control and Impact) at each of two marsh sites Middlebridge (North Middlebridge) and Canonchet (Star Drive). Within each Excel File the first sheet is the Canonchet site and the second sheet is the Middlebridge Site.
- 5 transects at each Control and Impact location with 3 to 6 plots along the transects.
- Number of plots at each location: Canonchet Control 22 plots and Impact 21 plots; Middlebridge Control 24 plots and Impact 23 plots. *Some planned Impact plots ***did not get runneled***: Middlebridge: Impact plots at T4 and T5 are called “NR” not runneled, Canonchet: Impact plots T1-75, 100, 125, and T2 -100 are called “NR” not runneled.

**Dataset: Vegetation Data**

*Description:*

- Collected using the point intercept method at 50 points within each plot
- Data was converted to percent cover (out of 100%)
- Species richness = number of plant species per plot
- Shannon Diversity Index =
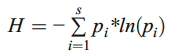


*Excel sheet headers:*

- Plot = Plot along transect:

“T1_” = transect number,

“_00” = plot along transect

- Trt = Treatment:

“C” = Control

“I” = Impact

“NR” = “not runneled”

- Columns E to BJ = Headers are species names abbreviated; data are percent cover of each species. Full species names associated with the codes are in the first sheet of the file called “Plant Species Codes”
- Columns BK to BU = Headers are other ground cover types; data are percent cover of each ground cover
- Sp_Rich = Summed number of plant species
- Shan_Div = Shannon Diversity Index for vegetation species

**Dataset: Groundwater Data**

*Description:*

- Collected within the channel and at 2 groundwater wells along the transects in each of the Control and Impact locations.
- Pressure data loggers used to calculate water level elevation
- Units are meters above sea level (NAVD standard)

*Excel sheet headers:*

- “Water_” = Level of water in the ground water well:
- “Ref_” = Level of the marsh plane at that site:

“_T1” = Transect number

“_45” = Plot along transect

“_I” = Impact site, “_C” = Control site

*Note: at Middlebridge plot 36 switches to plot 48 in 2017

**Dataset: Groundwater Salinity Data**

*Description:*

- Canonchet (at Star Drive) and Middlebridge sites collected yearly 2014-2019.
- Measured salinity in groundwater wells
- Two methods of measuring salinity: some salinity measured on samples extracted from the wells or measured within the wells.
- *Some planned Impact plots ***did not get runneled***: Middlebridge: Impact plots at T4 and T5 are called “NR” not runneled.

*Excel sheet headers:*

- Plot = Plot along transect:

“T1_” = transect number,

“_00” = site along transect

- Trt = Treatment:

“C” = Control

“I” = Impact

“NR” = Not Runneled

- Time_lowtide = Time on that date that low tide occurred
- Since_LowTide = Time since low tide occurred (units are hours); positive is hours after time of low tide, negative is hours before low tide
- Water_table = Water table depth (cm); positive is above marsh surface, negative is below marsh surface
- Salinity_extacted = salinity measured on extracted samples (units ppt)
- Salinity_in = alternative measurement of salinity (units ppt)
- Salinity = combined data from the two methods of measuring salinity, duplicate salinities were averaged (units ppt)

**Dataset: Groundwater Tides Data**

*Description:*

- Based the groundwater level dataset, this file has isolated the times and levels of the high and low tides in the channel and groundwater well data. The sheets in the document are for each of the two sites (Canonchet and Middlebridge) and years 2014-2018.
- R Package VulnToolkit (Hill and Anisfeld, 2021) was used to extract the times and water level at high and low tides in the channel each day. The groundwater level at the time of the high and low tides were also extracted.

*Excel sheet headers:*

- Time_High= Timing of the high tide level in the channel on that day
- Channel_High= Level of the water in the channel at high tide
- Ex. “T1_40_C”_High= Level of groundwater at high tide in each of the 4 wells
- Time_Low= Timing of the low tide level in the channel on that day
